# Natural Killer Cell Immune Checkpoints and Their Therapeutic Targeting in Cancer Treatment

**DOI:** 10.34133/research.0723

**Published:** 2025-06-03

**Authors:** Anqi Lin, Pengxi Ye, Zhengrui Li, Aimin Jiang, Zaoqu Liu, Quan Cheng, Jian Zhang, Peng Luo

**Affiliations:** ^1^ Donghai County People’s Hospital—Jiangnan University Smart Healthcare Joint Laboratory, Donghai County People’s Hospital (Affiliated Kangda College of Nanjing Medical University); Department of Oncology, Zhujiang Hospital, Southern Medical University, Lianyungang 222000, China.; ^2^ School of Traditional Chinese Medicine, Southern Medical University, Guangzhou, Guangdong 510515, China.; ^3^ Department of Oral and Cranio-Maxillofacial Surgery, Shanghai Ninth People’s Hospital, College of Stomatology, Shanghai Jiao Tong University School of Medicine, National Clinical Research Center for Oral Diseases, Shanghai Key Laboratory of Stomatology and Shanghai Research Institute of Stomatology, Shanghai 200011, China.; ^4^ Department of Urology, Changhai Hospital, Naval Medical University (Second Military Medical University), Shanghai, China.; ^5^ Institute of Basic Medical Sciences, Chinese Academy of Medical Sciences and Peking Union Medical College, Beijing 100730, China.; ^6^ Department of Neurosurgery, Xiangya Hospital, Central South University, Changsha, Hunan 410008, China.; ^7^ National Clinical Research Center for Geriatric Disorders, Xiangya Hospital, Central South University, Hunan, China.; ^8^ Department of Oncology, Zhujiang Hospital, Southern Medical University, Guangzhou, Guangdong 510282, China.

## Abstract

Natural killer (NK) cells, serving as pivotal mediators of innate immunity, play an important role in antitumor immunity. Immune checkpoint can be expressed on the surface of NK cells and meticulously regulates their activation states and effector functions through complex signaling networks. In recent years, tumor immunotherapy strategies focusing on NK cell immune checkpoints have demonstrated remarkable advancements. This review systematically elucidates the expression profiles, signaling pathways, and the immune checkpoint molecule regulatory mechanisms localized on the NK cell membrane (e.g., NKG2A, KIRs, and TIGIT) or intracellularly (e.g., BIM, Cbl-b, and EZH2) during tumor immune evasion. Particular attention is devoted to dissecting the regulatory mechanisms through which these immune checkpoint molecules influence NK cell-mediated cytotoxicity, cytokine secretion, proliferative capacity, and tunable modulation of NK cell immune checkpoint expression by diverse factors within the tumor microenvironment. Furthermore, this review comprehensively summarizes preclinical advancements in NK cell immune checkpoint blockade strategies, including single checkpoint blockade, combinatorial checkpoint approaches, and their integration with conventional therapeutic modalities. Additionally, emerging therapeutic advancements, such as gene-editing technologies and chimeric antigen receptor-NK (CAR-NK) cell therapy, are evaluated for their prospective applications in immunotherapy based on NK cells. By thoroughly elucidating the molecular regulatory networks underlying NK cell immune checkpoints and their mechanisms of action within the complex tumor microenvironment, this review aims to provide critical theoretical insights and translational foundations to foster the development of innovative tumor immunotherapy strategies, improvement of combination therapies, and realization of personalized precision medicine.

## Introduction

Immune checkpoint (IC) molecules represent a vital group of immunoregulatory molecules. These molecules effectively maintain immune system homeostasis. When activated, IC molecules function to modulate immune responses at appropriate levels, preventing excessive immune activation. IC molecules mediate costimulatory or co-inhibitory signals through receptor–ligand interactions, thereby regulating the functional states of immune cells during inflammatory reactions and immune tolerance. This mechanism prevents autoimmune reactions while maintaining a delicate equilibrium between pro-inflammatory and anti-inflammatory immune actions. In the tumor microenvironment (TME), tumor cells evade immune surveillance by expressing inhibitory ligands for IC molecules, which activates inhibitory signaling pathways in immune cells. This cascade induces immune cell dysfunction and creates a favorable environment for tumor cell survival [1,2]. In cancer immunology research, IC molecules have garnered marked attention, particularly T cell-associated IC molecules such as programmed cell death ligand-1 (PD-L1) and cytotoxic T lymphocyte-associated protein 4 (CTLA-4), among others. These molecules serve as critical mediators of tumor immune evasion. For instance, cancer cells expressing PD-L1 interact with programmed death receptor-1 (PD-1) on T cell surfaces, delivering immunosuppressive signals that induce T cell exhaustion [3]. Beyond T cells, researchers have identified several IC molecules on natural killer (NK) cells, including natural killer group 2 member A (NKG2A), killer cell immunoglobulin-like receptors (KIRs), and T cell immune receptor with Ig and ITIM domains (TIGIT), which possess significant clinical therapeutic potential [4]. Signals transduced by these receptors are integrated to regulate NK cell functional states, ultimately determining both NK cell-mediated cytotoxicity and cytokine secretion magnitude [5]. Extensive research is currently focused on IC molecules, particularly in cancer immunotherapy [1,6].

NK cells are key effector cells in tumor immunity [7]. In humans, NK cells are primarily defined by their CD3^−^CD16^+^CD56^+^ phenotype. Based on neural cell adhesion molecule expression levels, NK cells are subdivided into the CD56^bright^ (CD16^dim^) subset, associated with immunoregulatory functions, and the CD56^dim^ (CD16^+^) subset, associated with cytotoxic functions. Among these, the CD56^bright^ subset constitutes approximately 10% of peripheral blood NK cells (pNKs), representing a relatively immature yet highly proliferative population. In contrast, the CD56^dim^ subset, which accounts for approximately 90%, displays heightened cytolytic activity [8]. Notably, studies have identified an intermediate CD56^dim^CD16^dim^ subset exhibiting phenotypic and functional characteristics intermediate between these 2 subsets, both in healthy individuals and in patients with antigen-processing deficiencies [9]. The development, maturation, and functional regulation of NK cells are critically dependent on the transcription factors T-box expressed in T cell (T-BET) and eomesodermin (EOMES) [4]. Meanwhile, they are tightly regulated by multiple cytokines, including interleukin (IL)-2/4/7/12/15/18, at various stages of the immune response [3,10]. In the TME, the down-regulation of major histocompatibility complex class I (MHC-I) molecules on tumor cells can activate NK cells [3]. Additionally, NK cells are capable of secreting chemokines such as C-C motif chemokine ligand 5 (CCL5), X-C motif chemokine ligand 1 (XCL1), and X-C motif chemokine ligand 2 (XCL2) to recruit conventional type 1 dendritic cells (cDC1) to tumor sites, promote their differentiation and survival through FLT3 ligand, and synergistically contribute to the enlistment and stimulation of CD8^+^ T cells [4]. These functions make NK cells critical for combating early tumorigenesis and micrometastasis [3].

IC molecules on NK cell surfaces can be divided into 2 main categories: inhibitory and activating [7]. Inhibitory molecules comprise KIRs—initially identified and termed p58 molecules by Alessandro Moretta in 1990 [10]—and the CD94/NKG2A heterodimer, which belongs to the natural killer receptor 2 family that engages with MHC-I molecules [7]. Additionally, NK cells express other inhibitory molecules, including NKG2A, KIRs, TIGIT, and leukocyte immunoglobulin-like receptor B1 (LILRB1) [4]. The interaction between MHC-I molecules and inhibitory receptors on NK cell surfaces maintains NK cell quiescence. Consequently, reduced expression of MHC-I molecules on cell surfaces results in insufficient inhibitory signaling to NK cells [7]. The primary activating receptors on NK cells include natural killer group 2 member D (NKG2D), DNAX accessory molecule-1 (DNAM-1), and natural cytotoxicity receptors (NCRs), such as natural killer p30 (NKp30), natural killer p44 (NKp44), and natural killer p46 (NKp46). These activating receptors recognize specific ligands, generally absent on healthy cell surfaces, thereby promoting NK cell activation, initiating target cell lysis, and inducing pro-inflammatory cytokine secretion [e.g., interferon-γ (IFN-γ) and tumor necrosis factor-α (TNF-α)], ultimately mediating antitumor immune responses [7,11]. Beyond membrane-bound ICs, researchers have identified cytoplasmic and nuclear ICs that regulate NK cell differentiation, development, proliferation, and metabolism [12]. Signal transduction downstream of NK cell ICs is mediated through cytoplasmic immunoreceptor tyrosine-based inhibitory motifs (ITIMs) and immunoreceptor tyrosine-based activation motifs (ITAMs). Typically, ITIMs are defined as V/I/LxYxxL/V [13] and are localized within the cytoplasmic domains of NK cell inhibitory receptors (such as NKG2A, TIGIT, and KIRs), while ITAMs are identified as (D or E)xxYxx(L or I)_X6-8_Yxx(L or I) [14] and are present in activating immune receptors (such as NKG2D, NKp30, and NKp46) or their adapter proteins [such as CD3ζ, FcεR1γ, and DNAX activating protein of 12 kDa (DAP12)]. Upon receptor–ligand binding, tyrosines within ITIMs or ITAMs undergo phosphorylation, which leads to the recruitment of SH2 domain-containing protein phosphatases [such as src homology 2 domain-containing phosphatase-1 (SHP-1) and src homology 2 domain-containing phosphatase-2 (SHP-2)] or tyrosine kinases [such as spleen tyrosine kinase (Syk) and zeta-chain-associated protein kinase 70 (ZAP-70)], respectively, resulting in inhibition or activation of NK cell signaling pathways that ultimately modulate their antitumor functions [13–21].

Recent investigations have elucidated the functional significance of multiple IC molecules, including PD-1, TIGIT, and NKG2A, in NK cells and their potential therapeutic applications, as these molecules mediate NK cell functional exhaustion [5]. Here, we systematically review the expression profiles of both membrane-bound and intracellular IC molecules in NK cells, their associated signaling cascades, and their regulatory roles in tumor immune escape mechanisms. This review specifically addresses the mechanistic pathways through which NK cell IC molecules modulate cytotoxicity, cytokine secretion, and proliferative capacity. Moreover, we provide a comprehensive analysis of preclinical advances in NK cell IC blockade strategies, encompassing single-agent interventions, synergistic multi-checkpoint inhibition approaches, and combination therapies with conventional treatment modalities. Furthermore, we examine cutting-edge therapeutic approaches, including genome editing technologies and chimeric antigen receptor (CAR)-NK cell therapies within the context of NK cell-based immunotherapy. A deeper understanding of NK cell IC molecular regulatory networks and their functions within the complex TME will establish a fundamental theoretical framework and translational foundation for developing novel immunotherapeutic strategies, optimizing combination treatments, and advancing personalized precision medicine.

## ICs in NK cells

**Fig. 1.**
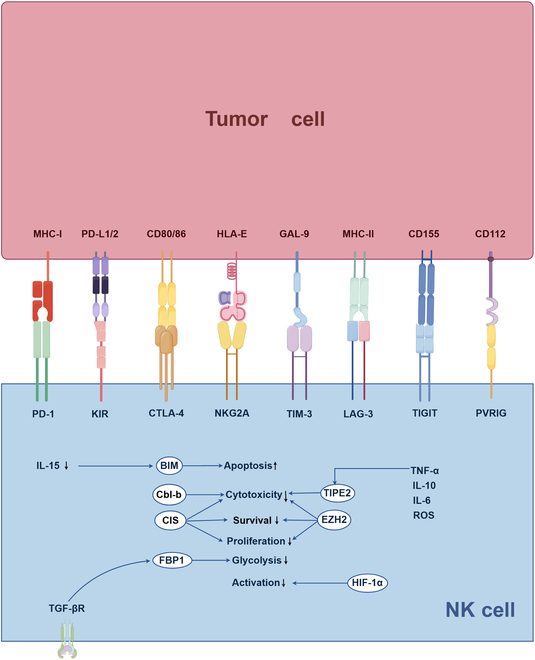
ICs expressed by NK cells. NK cells express a series of IC molecules both on their surface and within their interiors, which play essential roles in regulating their functions. Inhibitory receptors located on the NK cell membrane interact with ligands presented on the surface of tumor cells, thereby initiating biological processes that suppress NK cell activity, promote immune tolerance, and diminish antitumor immunity. Conversely, activating receptors have the ability to enhance NK cell activity. IC molecules residing within NK cells regulate intracellular signaling pathways, thereby affecting processes such as proliferation, metabolism, and apoptosis, which collectively influence their antitumor capabilities. The figure was drawn by Figdraw (www.figdraw.com). NK, natural killer; MHC, major histocompatibility complex; PD-L1/2, programmed cell death ligand 1/2; CD, cluster of differentiation; HLA, major histocompatibility complex; GAL, galectin; PD-1, programmed cell death protein 1; KIR, killer immunoglobulin-like receptors; CTLA-4, cytotoxic T lymphocyte-associated antigen-4; NKG2A, natural killer group 2 member A; TIM-3, T cell immunoglobulin domain and mucin domain-3; LAG-3, lymphocyte activation gene-3; TIGIT, T cell immune receptor with Ig and ITIM domains; PVRIG, poliovirus receptor-related immunoglobulin domain-containing protein; BIM, Bcl-2 interacting mediator of cell death; Cbl-b, Casitas B lineage lymphoma proto-oncogene b; CIS, cytokine-inducible SH2-containing protein; FBP1, fructose-1,6-bisphosphatase; EZH2, enhancer of zeste homolog 2; TIPE2, TNF-α-induced protein 8-like 2; HIF-1α, hypoxia-inducible factor-1α; IL, interleukin; TGF, transforming growth factor; ROS, reactive oxygen species; TNF, tumor necrosis factor.

IC molecules in NK cells serve as critical molecular switches that precisely regulate their biological functions and can be classified into 2 main categories based on their subcellular localization characteristics: membrane surface and intracellular checkpoints (Fig. 1). Membrane surface ICs bind to their specific ligands, thereby mediating the activation or inhibition of downstream signaling pathways and precisely regulating NK cell proliferation, differentiation, and effector functions. Intracellular ICs regulate NK cell functions through multiple mechanisms, including intracellular signal transduction networks, metabolic reprogramming, and epigenetic modifications [22]. This section systematically examines the structural characteristics, expression regulation, ligand recognition mechanisms, functional significance, and relevant targeted therapeutic strategies associated with NK cell membrane surface and intracellular ICs.

### ICs on the cellular membrane

Based on the characteristics of their mediated signaling pathways, cell membrane-associated NK cell IC molecules can be categorized into 2 major groups: inhibitory receptors and activating receptors. Inhibitory receptors primarily include the KIR family, NKG2A/CD94, TIGIT, PD-1, T cell immunoglobulin and mucin domain-containing protein 3 (TIM-3), lymphocyte activation gene-3 (LAG-3), CD96, poliovirus receptor-related immunoglobulin domain protein (PVRIG), the sialic acid-binding immunoglobulin-like lectins (Siglec) family, immunoglobulin-like transcript 2 (ILT2)/LILRB1, and CTLA-4, among others. These inhibitory receptors predominantly possess cytoplasmic domains containing ITIMs, which function as docking sites for membrane-proximal phosphatases SHP-1/2, thereby attenuating activating receptor signaling and consequently suppressing NK cell activation [13]. In contrast, activating receptors (such as NKG2D, DNAM-1, and the NCR family) recruit adaptor molecules containing ITAMs upon ligand binding, thereby activating downstream signal transduction cascades and promoting NK cell activation. As key effector cells of the innate immune system, NK cells are emerging as critical targets for cancer immunotherapy research [23,24]. IC inhibitors (ICIs) primarily enhance NK cell antitumor effector functions by modulating immunoregulatory receptors expressed on the NK cell surface. These therapeutic agents effectively alleviate the immunosuppressive state mediated by the TME through specific blockade of inhibitory IC receptors, thereby promoting NK cell activation and enhancing antitumor immune responses. Here, we summarize clinical trials of NK cell ICIs with preliminary clinical results (Table) and those currently without results (Table S1). To date, the U.S. Food and Drug Administration (FDA) has approved multiple ICIs for clinical application, including nivolumab, pembrolizumab, and atezolizumab that target the PD-1/PD-L1 signaling pathway, ipilimumab and tremelimumab that target CTLA-4, and relatlimab that targets LAG-3. These immunotherapeutic agents have demonstrated significant clinical efficacy in the treatment of diverse malignancies, including melanoma, head and neck squamous cell carcinoma, non-small cell lung cancer (NSCLC), renal cell carcinoma, and hepatocellular carcinoma (HCC) [25–30].

**Table.** Clinical trials of NK cell immune checkpoint blockade with available research findings

	National clinical trial no.	Disease	Drugs	Phase	Status
NKG2A	NCT03341936	Squamous cell carcinoma of the head and neck	Nivolumab; lirilumab	II	Active, not recruiting
	NCT04307329	Breast cancer	Monalizumab; trastuzumab	II	Completed
	NCT04590963	Squamous cell carcinoma of the head and neck	Monalizumab; cetuximab	III	Active, not recruiting
	NCT03822351	Stage III non-small cell lung cancer	Durvalumab; oleclumab; monalizumab	II	Completed
	NCT02671435	Advanced solid tumors	Monalizumab; durvalumab; cetuximab	I/II	Active, not recruiting
	NCT02557516	Chronic lymphocytic leukemia	Monalizumab	I/II	Terminated
PD-1	NCT04927884	Advanced triple negative breast cancer	Sacituzumab	I/II	Terminated
	NCT02660034	Solid tumors	Tislelizumab; pamiparib	I	Completed
	NCT03532451	Bladder cancer	Nivolumab; lirilumab	I	Completed
	NCT03241927	Melanoma	Pembrolizumab	II	Terminated
TIGIT	NCT03563716	Non-small cell lung cancer	Atezolizumab; tiragolumab	II	Active, not recruiting
TIM-3	NCT02608268	Advanced malignancies	Sabatolimab; spartalizumab; 5-aza-2′-deoxycytidine	I/II	Terminated
	NCT03489343	Metastatic cancer	Sym023	I	Completed
	NCT04812548	Myelodysplastic syndrome	Sabatolimab; azacitidine; venetoclax	II	Terminated
KIR	NCT03341936	Squamous cell carcinoma of the head and neck	Nivolumab; lirilumab	II	Active, not recruiting
	NCT03347123	Solid tumors	Epacadostat; nivolumab; ipilimumab; lirilumab	I/II	Terminated
	NCT01592370	Hematologic malignancies	Nivolumab; ipilimumab; lirilumab	I/II	Completed
	NCT02399917	Myeloid leukemia	Azacitidine; lirilumab	II	Terminated
	NCT00552396	Multiple myeloma	1- 7 F9	I	Completed
	NCT01222286	Smoldering multiple myeloma	IPH 2101	II	Completed
	NCT01248455	Multiple myeloma	IPH 2101	II	Terminated
	NCT00999830	Multiple myeloma	IPH 2101	II	Completed
	NCT01687387	Acute myeloid leukemia	IPH 2102	II	Completed
	NCT02481297	Chronic lymphocytic leukemia	Lirilumab; rituximab	II	Completed
LAG-3	NCT02996110	Advanced cancer	Nivolumab; ipilimumab; relatlimab	II	Completed
	NCT02750514	Advanced cancer	Nivolumab; dasatinib; relatlimab	II	Terminated
	NCT02935634	Advanced gastric cancer	Nivolumab; ipilimumab; relatlimab	II	Completed
	NCT03489369	Lymphoma	Sym 022	I	Completed
	NCT03493932	Glioblastoma	Nivolumab; BMS-986016	I	Completed
	NCT03470922	Melanoma	Relatlimab; nivolumab	II/III	Active, not recruiting
	NCT03724968	Metastatic melanoma	Nivolumab; ipilimumab; relatlimab	II	Terminated
KIR+PD-1+CTLA-4	NCT01714739	Solid tumor	Lirilumab; nivolumab; ipilimumab	I/II	Completed
PD-1+TIGIT	NCT04952597	Limited stage small cell lung cancer	Ociperlimab; tislelizumab; pemetrexed	II	Completed
PD-1+LAG-3	NCT02061761	Hematologic neoplasms	Relatlimab; nivolumab	I/II	Completed
PD-1+LAG-3	NCT04634825	Head and neck cancer	Enoblituzumab; retifanlimab; tebotelimab	II	Terminated
PD-1+LAG-3	NCT03484923	Melanoma	PDR001; LAG525; INC280	II	Completed
PD-1+LAG-3	NCT03662659	Gastric cancer	Nivolumab; relatlimab	II	Completed
PD-1+LAG-3	NCT03365791	Small cell lung cancer	PDR001; LAG525	II	Completed
PD-1+LAG-3	NCT03250832	Neoplasms	TSR-033; dostarlimab; mFOLFOX6;	I	Completed
PD-1+CTLA-4+LAG-3	NCT02488759	Various advanced cancer	Nivolumab; ipilimumab; relatlimab; daratumumab	I/II	Completed

NKG2A, natural killer group 2 member A; PD-1, programmed death-1; TIGIT, T cell immunoreceptor with immunoglobulin and immunoreceptor tyrosine-based inhibitory motif domains; TIM-3, T cell immunoglobulin and mucin-domain containing-3; KIR, killer cell immunoglobulin-like receptor; LAG-3, lymphocyte activation gene-3; CTLA-4, cytotoxic T lymphocyte antigen-4

#### Inhibitory receptors

##### KIR family

KIR (CD158) is a killer cell immunoglobulin (Ig)-like receptor whose gene family is located in the 19q13.4 chromosomal region, where each KIR gene spans approximately 10 to 16 kb and is arranged in a tightly linked head-to-tail configuration. KIRs exhibit significant structural diversity and functional polymorphism, and are classified into 2 major categories: activating and inhibitory [31]. This diversity is primarily manifested in the number of extracellular Ig-like domains (KIR2DL1 versus KIR3DL1), the length of cytoplasmic tails (KIR2DL1 versus KIR2DS1), and in sequence variations (KIR2DL1 versus KIR2DL3). Each KIR molecule specifically recognizes human leukocyte antigen (HLA) class I molecules (HLA-A, HLA-B, or HLA-C) as its ligands [25,32]. The interaction between KIR and its ligands regulates NK cell self-tolerance and the cytotoxic effects of these cells against transformed cells [33]. Inhibitory KIRs suppress NK cell functional activity by binding to MHC-I molecules [23]. Activating KIRs (aKIRs) are characterized by the absence of ITIM motifs in their cytoplasmic tails and the presence of transmembrane domains with positively charged amino acid residues [34], which enable them to associate with signal-transducing adaptor proteins KARAP/DAP12 containing ITAMs [35]. The most extensively studied aKIR is KIR2DS1, which recognizes HLA-C C2 allotypes, albeit with lower affinity than KIR2DL1 [35]. Experimental data indicate that despite high expression of KIRs in NK cells and tumor tissues, KIR-targeted therapies have demonstrated poor efficacy with frequent treatment escape phenomena, suggesting that gene editing at KIR loci may not yield ideal therapeutic results [31]. Research have shown that the expression levels of KIRs on pNKs are elevated in patients with endometriosis [36]. Due to the high variability of the KIR family, NK cells from different individuals may express either multiple polymorphic receptors or only specific subsets from the receptor repertoire, potentially leading to significant differences in the efficacy of KIR-targeted therapeutic antibodies among different individuals [25].

KIR3DL2. KIR3DL2 is one of the conserved framework genes in the KIR family, which is widely expressed in human populations, and functions as a recognized receptor for MHC-I molecules. Recent studies employing surface plasmon resonance technology and Fc fusion proteins that express various KIR family members have confirmed that immunoglobulin superfamily member 8 (IGSF8) specifically interacts with NK cells. Researchers have further verified that IGSF8 inhibits NK cell-mediated cytotoxic activity through specific binding to KIR3DL2, as demonstrated by analysis of NK cell degranulation markers via flow cytometry. Additionally, studies have demonstrated that KIR3DL2 exhibits high binding affinity to both IGSF8 and the anti-KIR3DL2 monoclonal antibody lacutamab [23].

KIR2DL5. KIR2DL5 is a significant member of the human KIR family that exhibits specific interactions with the poliovirus receptor (PVR). Research has demonstrated that when PVR is depleted from the tumor cell surface, the KIR2DL5-mediated inhibition of NK cell cytotoxicity is abrogated. Notably, the binding site of KIR2DL5 to PVR differs from those of other receptors such as DNAM-1, TIGIT, and CD96; consequently, KIR2DL5 does not compete with these receptors for PVR binding. Monoclonal antibodies that have been successfully developed block the interaction between KIR2DL5 and PVR in multiple humanized tumor models, providing novel therapeutic strategies for cancer immunotherapy [37,38].

Lirilumab (IPH2102, derived from 1-7F9) is a monoclonal antibody specifically targeting inhibitory KIRs (KIR2DL1 and KIR2DL2/3). The specific epitope recognized by this antibody is located in the first domain of KIR2DL3, spatially overlapping with the HLA molecule binding site (approximately 134 μ^2^), thereby effectively inhibiting the KIR-HLA signaling pathway. Because its epitope is completely masked by the third Ig-like domain of KIR3D receptors, lirilumab does not bind to KIR3D receptors. Preclinical studies have demonstrated that the combined application of lirilumab with IL-2-activated HLA-matched NK cells significantly enhances the in vitro lysis of patient-derived acute myeloid leukemia (AML) blasts. Although preliminary studies confirmed its safety profile, subsequent trial results indicated that the therapeutic efficacy of lirilumab was not significantly superior to standard treatment regimens, potentially due to KIR-HLA genotype mismatches in patients and the presence of other dominant inhibitory signals [39]. However, engineered versions of lirilumab and other anti-KIR antibodies with altered affinities and KIR specificities still possess significant development potential, warranting further exploration in preclinical investigations [25]. Nevertheless, KIR blockade therapy also faces challenges, as evidenced by a phase II clinical trial evaluating KIR2D checkpoint inhibitors in multiple myeloma (MM) patients that failed to demonstrate clinical benefit as monotherapy. Based on negative results from clinical trials in refractory MM patients, the therapeutic potential of anti-KIR antibody monotherapy in the treatment of malignancies appears to be limited [40].

##### NKG2A/CD94

NKG2A is an inhibitory receptor that is expressed on the surface of NK cells and T cells, and belonging to the C-type lectin family of heterodimeric receptors, it significantly inhibits NK cell activity [31]. NKG2A exerts its biological function by binding to CD94 molecules to form NKG2A/CD94 heterodimeric complexes [41]. This complex specifically recognizes the human nonclassical MHC-I molecule HLA-E and the mouse Qa-1b molecule (encoded by the H2-T23 gene), thereby making it a potential therapeutic target in both species [42]. The inhibitory function of NKG2A is regulated by different peptides presented by HLA-E molecules, specifically with signal peptides derived from HLA-B8 and HLA-A2, which generate strong interactions with NKG2A when bound to HLA-E. HLA-E complexes can significantly affect the therapeutic efficacy of anti-NKG2A antibodies by competitively interfering with the interaction between these antibodies and NKG2A [43]. Notably, NKG2A shares the HLA-E ligand with its activating homologous receptor natural killer group 2 member C (NKG2C), which also forms heterodimers with CD94. In individuals with prior cytomegalovirus infection, NKG2C^+^ NK cell populations demonstrate enhanced cytolytic activity and undergo significant population expansion. Research indicates that NKG2A has at least 6-fold higher affinity for HLA-E compared to NKG2C, resulting in more pronounced ligand-mediated cytotoxic inhibition when NKG2A is expressed [44].

Monoclonal antibodies targeting NKG2A. Monalizumab is a humanized monoclonal antibody targeting NKG2A, with a mechanism of action that specifically blocks the interaction between NKG2A on NK cell surfaces and HLA-E molecules highly expressed on tumor cells, thereby activating antitumor immune responses mediated by NK cells and cytotoxic T lymphocytes (CTLs). Clinical trial results have demonstrated that monalizumab exhibits promising therapeutic potential, especially when used in combination with other drugs such as durvalumab (anti-PD-L1 antibody), trastuzumab (anti-HER2 antibody), or cetuximab [anti-epidermal growth factor receptor (EGFR) antibody]. This multitarget combination therapy strategy not only synergistically enhances the therapeutic efficacy of ICIs but also more effectively prevents and overcomes tumor immune resistance by simultaneously activating both innate and adaptive immune systems. Although monalizumab monotherapy has not yet shown clear clinical benefits [44,45], breakthrough progress has been achieved in multiple combination therapy clinical trials. For example, when monalizumab is combined with the anti-PD-L1 monoclonal antibody durvalumab for the treatment of patients with advanced colorectal cancer (CRC), disease remission and stabilization have been observed in some patients. Furthermore, in phase III clinical trials, the combination regimen of monalizumab with the EGFR inhibitor cetuximab has also demonstrated significant clinical efficacy in patients with squamous cell carcinoma [9].

Novel therapeutic strategies targeting NKG2A. Multiple novel therapeutic strategies targeting NKG2A have been developed, such as the tyrosine kinase small-molecule inhibitor dasatinib that selectively down-regulates NKG2A expression levels, thereby enhancing NK cell antitumor activity while maintaining the expression levels of other inhibitory receptors. Currently, dasatinib has been approved as a first-line treatment for chronic myeloid leukemia (CML) [46]. CRISPR-Cas9 gene-editing technology can block inhibitory signal transduction in human primary NK cells by specifically targeting the KLRC1 gene (encoding NKG2A). Research indicates that deletion of NKG2A can significantly inhibit the phosphorylation levels of its downstream effector molecules SHP-1 and SHP-2, thereby demonstrating superior efficacy compared to traditional anti-NKG2A antibody blocking methods [47]. The CRISPR-Cas9 gene-editing approach developed by research teams can achieve approximately 80% knockout efficiency of the KLRC1 gene in primary NK cells, and studies have demonstrated that deletion of the KLRC1 gene significantly enhances NK cell cytotoxicity against MM cells [48]. CRISPR-mediated KLRC1 gene editing not only targets the NKG2A/HLA-E IC but also enhances NK cell cytotoxic responses against HLA-E-positive tumor cells by augmenting NKG2C function [49].

##### TIGIT

TIGIT, also known as WUCAM, VSTM3, or VSIG9, is a transmembrane protein containing an Ig domain and an ITIM. TIGIT is expressed on the surface of various immune cells, including CD4^+^ T cells, CD8^+^ T cells, NK cells, regulatory T cells (Tregs), and tumor-infiltrating lymphocytes (TILs) [50]. Studies have demonstrated that the expression pattern of TIGIT differs significantly from other IC molecules (such as PD-1, CTLA-4, and LAG-3): TIGIT is highly expressed in CD56^dim^CD16^dim^ NK cell subsets, while its expression level is significantly lower in CD56^bright^CD16^−^ NK cells [51]. As a critical IC molecule, TIGIT simultaneously regulates the survival and functional exhaustion of both NK cells and T cells [50]. TIGIT is a transmembrane protein composed of 244 amino acids encoded by the TIGIT gene located on chromosome 3q13.31, with its structure containing domains homologous to the PVR/nectin protein family [52]. Structurally, TIGIT comprises 3 major domains: an extracellular immunoglobulin variable set (IgV) domain, a type I transmembrane domain, and a highly conserved intracellular inhibitory domain containing both ITIM and immunoglobulin tyrosine tail (ITT) motifs. Experimental evidence indicates that the inhibitory function of murine TIGIT is mediated through phosphorylation of ITIM tyrosine residues (Y277) or ITT-like motif residues (Y233). TIGIT expression levels positively correlate with various immune response-related inflammatory factors, including interferons, MHC-I and MHC-II, lymphocyte-specific protein tyrosine kinase (LCK), hematopoietic cell kinase (HCK), and signal transducer and activator of transcription 1 (STAT1) [52]. TIGIT interacts with multiple ligands, including CD155 (PVR or Necl-5), CD112 (PVRL2, nectin-2), CD113 (PVRL3, nectin-3), and nectin-4 (PVRL4, PRR4), with CD155 serving as its primary high-affinity ligand.

TIGIT blockade represents a potential therapeutic target for reversing T cell and NK cell functional defects [53]. Vibostolimab, a highly specific TIGIT-targeted monoclonal antibody, selectively inhibits the interaction between TIGIT and its ligands CD112 and CD155. However, single-blockade strategies demonstrate limited therapeutic efficacy in certain diseases; therefore, combining multiple IC receptor targets to achieve synergistic effects represents a more effective therapeutic approach [53]. A study on castration-resistant prostate cancer (CRPC) demonstrated that the combined administration of TIGIT monoclonal antibodies and allogeneic NK cell therapy not only more potently eradicates CRPC cells but also converts “cold” TMEs into “hot” TMEs at lower NK cell doses, a finding with significant clinical translational value [54]. In the MORPHEUS-Liver study (NCT04524871), researchers evaluated a triple combination therapy regimen consisting of anti-PD-L1 monoclonal antibody (atezolizumab), anti-vascular endothelial growth factor monoclonal antibody (bevacizumab), and anti-TIGIT monoclonal antibody (tiragolumab) for patients with unresectable locally advanced or metastatic HCC, which significantly prolonged progression-free survival (PFS) [50]. In patients with chronic hepatitis B virus (HBV) infection, the combined administration of IL-21 and anti-TIGIT antibodies promotes IFN-γ secretion by CD56^dim^ NK cells and enhances the clearance of HBsAg and HBeAg through splenic NK cells, with this combination therapy approach exhibiting more significant therapeutic effects compared to single TIGIT pathway blockade [55].

##### PD-1

PD-1 (CD279) is a type I transmembrane protein encoded by the PDCD1 gene, which belongs to the expanded members of the CD28/CTLA-4 Ig superfamily [56,57]. The PD-1 protein consists of 288 amino acids, featuring a structure that includes an extracellular Ig-V-like N-terminal domain, a hydrophobic transmembrane region, and an intracellular cytoplasmic tail. The cytoplasmic tail contains 2 key tyrosine phosphorylation sites: an ITIM and an immunoreceptor tyrosine-based switch motif (ITSM) [57]. PD-1 is expressed on various immune cells, including T cells, B cells, monocytes, dendritic cells, and NK cells, playing important physiological functions in maintaining peripheral immune tolerance [56,58]. Research has demonstrated that PD-1 expression is NK cell subset-specific, being expressed only on CD56^dim^ NK cells but not on CD56^bright^ NK cells, and is primarily limited to terminally differentiated NK cell subsets with NKG2A^−^KIR^+^CD57^+^ phenotypes [59]. Notably, PD-1 expression is restricted to NK cells within the TME rather than across all NK cell populations [60]. Further studies indicate that PD-1 on the NK cell surface is not endogenously expressed but is instead acquired through phagocytosis of tumor cells, a process mediated by signaling lymphocytic activation molecule receptors. Under normal physiological conditions, NK cells derived from healthy donors do not express PD-1 protein on their cell surface. However, when NK cells are cocultured with tumor cells, during the process of cell contact and cytotoxic granule release, PD-1 protein is transferred to the NK cell membrane surface, resulting in increased membrane surface PD-1 expression levels, which is accompanied by surface aggregation of CD107a molecules. PD-1 acquired through this phagocytic pathway can inhibit the antitumor immune function of NK cells, a finding that provides an important theoretical basis for the development of related monotherapy and combination immunotherapy strategies [61,62].

PD-1 has 2 major ligands, PD-L1 and PD-L2, which are aberrantly overexpressed in various tumors [63]. The binding of PD-1 to PD-L1 inhibits multiple key signaling pathways, including phosphatidyl-inositol,3,4,5 triphosphate (PIP3)–AKT–mechanistic target of rapamycin (mTOR), NK–extracellular signal-regulated kinase (ERK)–mitogen-activated protein kinase (MAPK), and STAT5 signaling pathways, ultimately leading to suppression of NK cell immune responses [9]. In the TME, the PD-1/PD-L1 pathway serves as a critical regulatory mechanism for inducing and maintaining immune tolerance [57], primarily exerting its immunosuppressive function by inhibiting the activation of the phosphatidylinositol 3-kinase (PI3K)/AKT signaling pathway in NK cells [64]. Notably, in addition to the PD-1/PD-L1 axis, PD-1/PD-L2 interactions also play a significant role in tumor immune evasion processes [10]. Tumor cells induce local immune tolerance by up-regulating PD-L1 expression, thereby inhibiting the antitumor activity of NK cells and T cells. PD-L1 expression is up-regulated by various inflammatory factors, with IFN-γ exhibiting the strongest inductive effect [56]. Although immunotherapy based on PD-1/PD-L1 blockade induces durable antitumor immune responses in a subset of patients, tumors may still develop therapeutic resistance through immune evasion mechanisms due to the selectivity of both adaptive and innate immune responses [65].

PD-1 blockade-related therapies. Currently, monoclonal antibodies targeting the PD-1/PD-L1 signaling pathway include nivolumab, pembrolizumab, and atezolizumab, all of which have demonstrated significant therapeutic efficacy in multiple clinical trials. Nivolumab has demonstrated significant clinical efficacy and favorable safety profiles in treating patients with relapsed/refractory CML and classical Hodgkin lymphoma [9]. For patients with metastatic CRC characterized by DNA mismatch repair deficiency or high microsatellite instability, nivolumab treatment can achieve durable disease remission and long-term survival benefits [66]. The phase III KEYNOTE-024 clinical trial further substantiated the clinical efficacy of PD-1 blockade therapy in the treatment of advanced NSCLC [67]. In a phase I clinical trial (NCT02964013) involving patients with advanced solid tumors, researchers observed that the combination regimen of pembrolizumab and the TIGIT monoclonal antibody vibostolimab significantly enhanced the immune activity of NK cells and CD8^+^ T cells in patients [52]. The combination regimen of nivolumab and the anti-KIR monoclonal antibody lirilumab elicited positive treatment responses in a phase II clinical trial for recurrent squamous cell carcinoma [9]. Additionally, the IGSF8.06 antibody targeting IGSF8 and NK cell-related receptors, when administered in combination with anti-PD-1 or anti-PD-L1 antibodies, can effectively inhibit immune evasion and therapeutic resistance resulting from tumor cells down-regulating antigen presentation [23]. A phosphorylated dendrimer polymer/anti-PD-1 nano-delivery system encapsulated with Mlm has also been developed, which demonstrates the capacity to penetrate the blood–brain barrier and enhances the immunotherapeutic efficacy against gliomas by synergistically regulating NK cell and T cell functions, offering novel approaches for the development of tumor immunotherapy strategies [68].

Combination therapeutic strategies of PD-1 blockade with other immunotherapies. Currently, combination therapy utilizing PD-1 blockade with other immunotherapies has demonstrated significant therapeutic effects against various tumors. In studies of AML, various combinations of IL-15, heat shock protein 70 (Hsp70), and PD-1 blockade have been found to significantly enhance NK cell-mediated cytotoxicity. Treatment with IL-15 combined with PD-1 blockade promotes the secretion of IFN-γ, perforin, and granzyme B, thereby enhancing the activation status and antitumor efficacy of NK cells in AML patients. Upon addition of the antigenic peptide Hsp70, a significant reduction in the proportion of PD-1-positive NK cells and their mRNA expression levels was observed, whereas PD-1 blockade simultaneously led to decreased expression of the inhibitory receptor NKG2A. These findings provide compelling evidence for AML treatment strategies based on cytokines and immunomodulators [69]. The combined application of the STAT3 signaling pathway small-molecule inhibitor YHO-1701 and PD-1/PD-L1 blockade significantly inhibits tumor growth in immunotherapy-resistant CMS5a fibrosarcoma mouse models, thus providing crucial experimental evidence for developing novel cancer immunotherapy combination strategies [70]. In aggressive cancer stem cell-like/poorly differentiated oral tumor models, combined treatment with PD-1 antibody and super-enhanced NK cells not only significantly augmented NK cell-mediated cytotoxicity but also stimulated IFN-γ secretion, thereby improving therapeutic efficacy [71]. Additionally, treatment strategies involving combinations of PD-1 blockade with the IL-33/ST2 signaling pathway, PD-1 blockade with EP4-targeted therapy, and TIGIT or PD-1 antibodies with ataxia telangiectasia and rad3-related protein inhibitor (ATRi)/radiation therapy (RT) have all demonstrated remarkable therapeutic potential [38,72,73].

##### TIM-3

TIM-3 is a member of the TIM receptor family that is expressed on the surface of various immune cells, including T cells, NK cells, and antigen-presenting cells, and participates in the regulation of key immune processes such as cell proliferation, survival, and tissue regeneration [74]. The extracellular region of TIM-3 contains a variable immunoglobulin-like (IgV) domain that specifically recognizes multiple ligands, including high mobility group box 1 (HMGB1), galectin-9, phosphatidylserine (PS), and carcinoembryonic antigen-related cell adhesion molecule 1 (CEACAM-1). Research has demonstrated that PS binding to TIM-3 induces its phosphorylation, subsequently inhibits the PI3K/mTORC1/p-S6 signal transduction pathway, and ultimately results in NK cell dysfunction [75]. The intracellular domain of TIM-3 consists of 5 highly conserved tyrosine residues, which interact with multiple components of the T cell receptor (TCR) complex [45].

##### LAG-3

LAG-3 (CD223) is expressed on the surface of activated NK cells, with its expression levels being up-regulated in response to IL-12 and IL-15 [31]. LAG-3 shares structural homology with CD4 but exhibits higher binding affinity for MHC-II molecules [45]. In the TME, the primary ligands for LAG-3 include liver sinusoidal endothelial cell lectin (LSECtin) and galectin-3. The interaction between LAG-3 and LSECtin inhibits effector T cell secretion of IFN-γ while simultaneously promoting TCR-mediated IL-10 production in melanoma. The LAG-3 signaling pathway directly inhibits initial T cell activation, whereas blocking LAG-3 on T cells enhances their proliferative capacity and cytokine secretion [1]. Notably, LSECtin, a member of the DC-SIGN family, is expressed not only in the liver but also in various tumor tissues, thereby functioning as an important potential ligand for LAG-3-positive immune cells [34]. Research indicates that LAG-3 synergistically inhibits antitumor immune responses with PD-1 through its structural similarity to CD4 and co-receptor properties, thereby not only suppressing CD4^+^ T cell function but also promoting Treg proliferation and up-regulating IL-10 expression, which collectively enhances the immunosuppressive TME.

##### CD96

CD96 is a type I transmembrane glycoprotein primarily expressed on the surface of T cells and NK cells, which competitively binds to the shared ligand CD155 with the costimulatory receptor CD226, exhibiting relatively high binding affinity [76]. Studies have found that the binding affinity of CD96 to PVR (CD155) falls between that of TIGIT and DNAM-1 [37,77], with the balance and competition among these 3 molecules serving as key factors in regulating NK cell functional activity. The up-regulation of CD96 expression is mediated by transforming growth factor-β1 (TGF-β1), primarily through the Smad family member 3 (SMAD3) signaling pathway [76]. CD96 was initially cloned and identified in human T cells, and its structure features 3 extracellular Ig domains and an ITIM motif, as well as a YXXM motif that can recruit the PI3K p85 subunit. Additionally, human CD96 can generate different isotype variants through alternative splicing, which present different extracellular domains, resulting in variations in its binding affinity to PVR [37,77]. CD96 was initially discovered to promote adhesion between NK cells and target cells, thus enhancing NK cell cytolytic function [37].

The CD96 ligand CD155 is highly expressed in various tumor tissues, including breast cancer, lung cancer, colon cancer, and pancreatic cancer [78]. Up-regulation of CD96 expression in tumor-infiltrating NK cells leads to NK cell dysfunction, which is associated with poor clinical prognosis. Notably, CD96 demonstrates significant species differences between mice and humans. In mice, CD96 expression levels are significantly higher than in humans, with almost all resting cells expressing CD96, while the basal expression level in human cells is relatively low [79]. Furthermore, the intracellular domain of mouse CD96 contains an ITIM motif, while the structural characteristics of human CD96 enable it to potentially function as both an activating and inhibitory receptor [80,81]. Among human NK cell subsets, CD96 expression also differs, with expression levels being significantly higher on CD56^bright^ NK cells than on CD56^dim^ NK cells [82].

##### PVRIG

PVRIG is a member of the Ig receptor superfamily, specifically belonging to the nectin and nectin-like protein (Necl) family. The primary ligand of PVRIG is PVRL2 (also known as CD112 or Nectin-2), and their interaction activates T cell inhibitory signaling pathways, resulting in functional impairment of TILs. Additionally, PVRIG inhibits T cell and NK cell functions by competitively binding to PVR and PVRL2 in competition with DNAM-1 (CD226) [83,84]. PVRIG exhibits higher binding affinity for CD112 compared to DNAM-1 or TIGIT, with its expression levels varying among different cell subpopulations. Under physiological conditions, approximately 5% to 15% of mouse NK cells express CD112R, whereas in human NK cells, PVRIG expression is detected in both CD16-positive and CD16-negative subpopulations. Unlike TIGIT and DNAM-1, PVRIG on NK cell surfaces undergoes internalization, thereby maintaining stable total cellular PVRIG levels. Studies have demonstrated that PVRIG is not expressed on other immune cells including B cells, monocytes, and neutrophils [84]. Studies have demonstrated that PVRIG is not expressed on other immune cells including B cells, monocytes, and neutrophils [84].

##### Siglec family

Siglecs are members of the type I lectin superfamily, which primarily function as immunoregulatory receptors. The majority of the currently identified members of the Siglec family possess inhibitory functions, including Siglec-2, Siglec-3, Siglec-5, Siglec-6, Siglec-7, Siglec-8, Siglec-9, Siglec-10, and Siglec-11, among others [32]. Siglecs function as a class of transmembrane surface receptors that are capable of specifically recognizing and binding to sialic acid-containing glycan structures (sialoglycans), thereby mediating intracellular activation or inhibitory signals [82]. Sialic acid represents a class of monosaccharides with a 9-carbon atom backbone, which serves as an important component at the terminals of glycoprotein and glycolipid molecules [85]. Human NK cells primarily express 2 Siglec receptors, Siglec-7 and Siglec-9, on their cell surface, both of which contain intracellular C-terminal regions with one or more ITIMs and ITIM-like sequences. Siglec-7 and Siglec-9 exhibit significant homology in both structure and function [85]. Siglec-7 (CD328, p75/AIRM-1) is expressed on the vast majority of pNKs in healthy individuals, while Siglec-9 expression is specifically limited to the CD56^dim^ NK cell subset [82].

In tumor-infiltrating NK cells, the expression level of Siglec-9, but not Siglec-7, is significantly up-regulated. Notably, in HCC patients, high expression of Siglec-9 is significantly associated with poor prognosis [86]. Studies have demonstrated that the proportion of Siglec-9-positive NK cells in the peripheral blood of patients with malignant melanoma and CRC is significantly lower compared to that in healthy control subjects. Further functional studies indicate that in healthy individuals, Siglec-9-positive cells within the CD56^dim^ NK cell subset exhibit notably lower cytotoxic activity against K562 target cells compared to their Siglec-9-negative counterparts. These results suggest that Siglec-9 may play a role in antitumor immune responses by regulating the cytotoxic function of NK cells [82]. Therefore, Siglec-9 represents a promising therapeutic target for cancer, as evidenced by existing studies wherein the small-molecule inhibitor MTX-3937 was shown to significantly enhance NK cell function and improve survival through targeting Siglec-9 and inhibiting the phosphorylation of both Siglec-9 and its downstream molecules SHP1 and SHP2. In NOD-Prkdcem26Cd52il2rgem26Cd22/Nju (NCG) mouse HCC xenograft models, MTX-3937 demonstrated significant antitumor efficacy [86].

##### ILT2/LILRB1

ILT2 (LILRB1/CD85j) belongs to the leukocyte Ig-like receptor (LIR/ILT) family and is highly expressed in NK cells. This receptor recognizes and binds to classical and nonclassical HLA-I, demonstrating a higher affinity for the nonclassical HLA-G molecule, thereby exerting immunosuppressive functions. Upon ligand binding, ILT2 regulates the expression profile of various cytokines, inhibiting NK cell production of IFN-γ while up-regulating the expression of chemokines CCL2, CCL8, and C-X-C motif chemokine ligand 2/3 (CXCL2/CXCL3). Studies have demonstrated that CCL2 expression levels are significantly elevated in various tumor tissues and their TMEs, including CRC and esophageal squamous cell carcinoma (ESCC) [31,87]. Additionally, inhibitory interactions between LILRB1 on phagocyte surfaces and the β2-microglobulin subunit of MHC-I molecules on tumor cell surfaces may contribute to tumor cell resistance to phagocytosis [88].

##### CTLA-4

CTLA-4 (CD152) is a critical inhibitory transmembrane protein that comprises an extracellular receptor domain and an intracellular domain with 2 signal-transducing tyrosine motifs, exerting a negative regulatory role in NK cell activation [89,90]. The ligands of CTLA-4 include CD80 (B7-1) and CD86 (B7-2), which also serve as ligands for the T cell costimulatory molecule CD28, which shares structural homology with CTLA-4. Notably, CTLA-4 exhibits significantly higher binding affinity and avidity for these 2 ligands compared to CD28, thereby enabling it to effectively antagonize CD28-mediated costimulatory signals [63]. CTLA-4 exerts its immunosuppressive functions through both intracellular and extracellular mechanisms: competing with CD28 for shared ligands, inhibiting downstream TCR signal transduction, transmitting inhibitory signals that affect T cell function, and mediating the clearance of B7-1 and B7-2 from antigen-presenting cell (APC) surfaces [91].

CTLA-4 is primarily expressed in Tregs and functionally exhausted T cells [57], and is also detectable on the surface of tumor-infiltrating NK cells, where its expression inhibits myeloid dendritic cell (mDC)-induced IFN-γ production [91]. Elevated CTLA-4 expression on Treg cell surfaces inhibits NK cell cytotoxic activity; consequently, CTLA-4 blockade enhances NK cell cytotoxic effects through both direct and indirect pathways. Research demonstrates that IL-15 up-regulates CTLA-4 expression on NK cell surfaces [90]. However, some studies have revealed that IL-15 selectively up-regulates CD28 but not CTLA-4 expression, while IL-2 has been established to induce CTLA-4 expression [91]. Although CTLA-4 is highly expressed in Tregs and serves a crucial role in conventional T cell self-tolerance, CTLA-4 blockade combined with Treg depletion strategies has demonstrated significant therapeutic efficacy in tumor treatment and autoimmune disease management [63].

Ipilimumab, a specific monoclonal antibody targeting CTLA-4, interacts with primary NK cells, IL-2-activated NK cells, and γδT cells via FcγRIIIA receptors, thereby triggering antibody-dependent cell-mediated cytotoxicity (ADCC) responses against CTLA-4-expressing melanoma cell lines and tissues [31]. Additionally, researchers have made significant progress in the field of NK cell immunotherapy, successfully developing various novel monoclonal antibodies, including the tri-specific NK cell engager antibody B7-H3xTIGITxCD16[92] and the humanized anti-PVRIG antibody IBI352g4a [83]. Recent studies have demonstrated that the novel IC molecule IGSF8 is expressed on tumor cell surfaces and forms specific interactions with KIR3DL2 receptors on human NK cell surfaces and Klra9 receptors in mice [23]. Based on this finding, researchers have developed the IGSF8.06 antibody, which specifically blocks the interaction between KIR3DL2 and IGSF8, thereby significantly enhancing NK cell antitumor activity [23]. In melanoma mouse models, the combined application of anti-CTLA-4 monoclonal antibody (ipilimumab) and IL-15 significantly enhances NK cell activation and improves their cytotoxic effects against B lineage acute lymphoblastic leukemia cell lines (Nalm-6) [90].

#### Activating receptors

##### NKG2D

NKG2D functions as a key activation receptor on NK cells. It initiates NK cell activation by recognizing specific ligands expressed on the surface of target cells, notably MHC-I polypeptide-related sequence A (MICA) and MHC-I polypeptide-related sequence B (MICB) [3]. In humans, 8 distinct ligands for NKG2D (NKG2DLs) have been identified to date. In addition to MICA and MICB, other ligands, which exhibit less than 25% homology to MICA/MICB, include the ULBP family, comprising ULBP-4 (RAET1E), ULBP-6 (RAET1L), ULBP-2 (RAET1H), ULBP-5 (RAET1G), ULBP-3 (RAET1N), and ULBP-1 (RAET1I) [93]. Soluble MICA (sMICA), a key ligand for NKG2D, promotes endocytosis and subsequent degradation of the receptor complex upon binding, thereby diminishing the surface expression of NKG2D. Certain tumor cells significantly impair NK cell function and promote tumor progression by secreting elevated levels of sMICA [56,94]. The soluble form of MICA (sMICA) has been detected in various malignancies, including lung, colorectal, gastric, liver, and breast cancers. Initial research suggested that MICA and MICB are scarcely detectable or absent in normal cells. However, subsequent studies have revealed that MICA and MICB transcripts are present in most normal tissues outside the central nervous system [93]. Moreover, NKG2D expression is crucial for antitumor immune responses, while its down-regulation represents a significant mechanism for tumor immune evasion [56].

##### DNAM-1

DNAM-1 (CD226) is a critical activating receptor on the surface of NK cells that mediates their activation by recognizing ligands such as Nectin-2 (CD112) and PVR (CD155), which are expressed on the surface of tumor cells [3]. Structurally, DNAM-1 consists of 2 tandemly arranged IgV domains and interacts with its ligand CD155 through a specific “double lock-and-key” mechanism [7]. DNAM-1 synergizes with other NK cell receptor ligands [including intercellular adhesion molecule-1 (ICAM-1), NKG2D-L, and CD48] to recognize CD155, thereby inducing NK cell cytotoxic responses that inhibit viral replication and control tumor burden [77]. DNAM-1 plays a pivotal role in NK cell-mediated tumor immunosurveillance, as studies have demonstrated the expression of DNAM-1 ligands CD155 and CD112 across a wide spectrum of solid tumors and hematologic malignancies. Notably, in NK cell-mediated antitumor cytotoxic responses, the blockade of CD155—but not CD112—significantly reduces NK cell cytotoxic efficacy, establishing CD155 as the primary DNAM-1 ligand involved in mediating NK cell antitumor activity [77]. Additionally, the interaction with CD155 dynamically modulates DNAM-1 expression on the surface of NK cells, enabling certain tumor cells to evade DNAM-1-mediated immune surveillance through distinct immune escape mechanisms, ultimately resulting in unfavorable clinical outcomes [77]. Beyond its interaction with DNAM-1, CD155 also binds to TIGIT. Studies have demonstrated that glycosylation at the N105 site of CD155 preferentially facilitates DNAM-1-mediated NK cell activation rather than TIGIT-mediated NK cell inhibition [95]. Mechanistic studies have revealed that elevated PVR expression in tumor cells, through binding to DNAM-1, induces tyrosine phosphorylation of DNAM-1’s cytoplasmic tail, which subsequently initiates its ubiquitin-dependent internalization and proteasomal degradation pathway [37]. DNAM-1-mediated cytotoxic activity in NK cells is regulated by a multifaceted molecular network, wherein NKG2D suppresses DNAM-1-mediated cytotoxicity, and TIGIT up-regulation amplifies this inhibitory effect, consequently promoting tumor immune evasion [96].

##### NCR receptors

The NCR family, including NKp30, NKp44, and NKp46, comprises pivotal molecules that mediate NK cell activation. NKp44 is a transmembrane glycoprotein encoded by the NCR2 gene; it is characterized by an IgV-type-like extracellular domain and lacks a homologous gene in the murine genome. NKp44 expression is predominantly confined to the CD56^bright^ NK cell subset. NKp44 mediates NK cell activation by engaging with the MHC-II molecule HLA-DP401 [7,97]. To date, the identified ligands for NKp44 include heparan sulfate, truncated isoforms of the mixed-lineage leukemia 5 protein, and soluble platelet-derived growth factor DD (PDGF-DD) [98]. The binding of NKp44 to PDGF-DD induces the secretion of TNF-α and IFN-γ, thereby both inhibiting tumor growth and promoting angiogenesis. Studies have demonstrated that NKp44 recognizes proliferating cell nuclear antigen (PCNA), a process predominantly mediated by spliced isoform 1 of NKp44. PCNA, functioning as a cancer-related nuclear factor, interacts with NKp44 to suppress NK cell functionality. A specific monoclonal antibody (mAb 14-25-9) can block the interaction between NKp44 and PCNA, thereby enhancing NK cell activity [99]. Notably, NKp44 also exhibits immunosuppressive properties, primarily through its specific interaction with PCNA, which triggers the NKp44/ITIM inhibitory pathway. The presence of PCNA on the tumor cell surface acts as an IC via the inhibitory axis involving NKp44-1 and ITIM, facilitating tumor cell evasion of NK cell-mediated immune clearance [98,100].

Natural cytotoxicity-triggering receptors NKp30 and NKp46 are constitutively expressed on most resting human NK cells. NKp46 specifically recognizes the calreticulin P domain, which becomes mislocalized to the cell surface during endoplasmic reticulum stress. NKp46 expression is negatively regulated through the TNF-α/TNF receptor 2 (TNFR2)/baculoviral IAP repeat containing 3 (BIRC3)/TNF receptor-associated factor 1 (TRAF1) signaling pathway.

NKp30 recognizes a diverse array of ligands, including B7-H6, which are expressed on tumor cells [101,102]. Upon binding to B7-H6, the transmembrane arginine residue of NKp30 engages with adaptor molecules containing ITAMs, such as the CD3ζ chain. This interaction subsequently triggers cytoskeletal rearrangement and calcium ion influx in NK cells, ultimately leading to the secretion of inflammatory cytokines. Studies have demonstrated that within the TME, tumor cells can be modulated using histone deacetylase (HDAC) inhibitors or small interfering RNA (siRNA) targeting HDAC2 and HDAC3. Such modulation diminishes B7-H6 expression on the surface of tumor cells, consequently impairing NKp30-dependent NK cell cytotoxicity [103]. NKp30 exists in 3 splice variants—NKp30a, NKp30b, and NKp30c. Among these variants, NKp30a and NKp30b exhibit activating functions, whereas NKp30c exerts immunosuppressive effects. All these isoforms recognize tumor-associated antigens, including B7-H6 and B cell lymphoma 2 (BCL-2) [104]. NKp30 function is further influenced by glycosylation modifications. Glycosylation at the N43 site enhances NKp30’s binding affinity to B7-H6 and stabilizes the B7-H6 protein. Conversely, glycosylation at the N208 site preserves the membrane localization of B7-H6, preventing its release as a soluble form. These glycosylation modifications modulate the interaction between NKp30 and B7-H6, ultimately influencing the cytotoxic function of NK cells [105].

### Intracellular ICs

NK cells express a variety of IC molecules within their intracellular and intramembranous regions, including Bcl-2 interacting mediator of cell death (BIM), Casitas B lineage lymphoma proto-oncogene b (Cbl-b), cytokine-inducible SH2-containing protein (CIS), enhancer of zeste homolog 2 (EZH2), fructose-1,6-bisphosphatase (FBP1), TNF-α-induced protein 8-like 2 (TIPE2), and hypoxia-inducible factor-1α (HIF-1α). These molecules regulate intracellular signaling networks via integration of signaling cascades triggered by various surface receptors. Consequently, they influence the proliferation, differentiation, and effector functions of NK cells [106–109].

#### BIM

BIM is a critical pro-apoptotic component within the Bcl-2 protein family [110] and functions as a pivotal regulator in the intricate network of programmed cell death pathways. BIM is essential for cytokine withdrawal-induced apoptosis of NK cells. Research has demonstrated that BIM-deficient NK cells exhibit reduced sensitivity to IL-15 withdrawal-induced apoptosis while preserving their cytotoxic activity and cytokine secretion capacity. The loss of the IL-15 signaling pathway results in the up-regulation of BIM expression [109]. BIM deficiency markedly improves immune cell survival and increases the number of NK cells, supporting the notion that BIM represents a promising therapeutic target in NK cell-mediated antitumor immunity [12].

#### Cbl-b

Cbl-b is a pivotal E3 ubiquitin ligase primarily interacting with its substrate proteins through its tyrosine kinase binding (TKB) domain, ubiquitin-associated (UBA) domain, and proline-rich (PR) region, and exerts its biological functions by mediating signaling protein ubiquitination [111]. Cbl-b specifically targets the transcription factor Foxp3 for ubiquitination through its synergistic interaction with the stress-related protein Stub1, thereby modulating the functions of thymic Tregs [112]. Studies have demonstrated that the loss of Cbl-b in NK cells markedly enhances their capacity for immune surveillance against tumor metastasis [113]. Genetic knockout of Cbl-b markedly enhances the cytotoxic efficacy of NK cells and increases the production of IFN-γ and the release of perforin, thereby augmenting their tumor cell-killing efficacy [12].

#### CIS

CIS is a member of the suppressor of cytokine signaling (SOCS) family whose expression is regulated by the IL-15 signaling pathway. CIS inhibits the Janus kinase (JAK)–STAT5 signaling pathway through a negative feedback mechanism [114,115]. In NK cells, CIS serves as a pivotal negative regulator of the IL-15 signaling pathway. Deletion of the CIS gene enhances the responsiveness of NK cells to IL-15, thereby promoting their proliferation and survival, increasing IFN-γ secretion, and boosting antitumor cytotoxic activity, ultimately improving the suppression of tumor metastasis [108]. Furthermore, the inhibition of CIS in conjunction with TGF-β inhibitors exerts a synergistic effect, further enhancing the antitumor activity of NK cells [116].

#### EZH2

EZH2 serves as the catalytic core subunit of the polycomb repressive complex 2 (PRC2) [117,118]. The C-terminal region of EZH2 contains a SET domain that mediates the trimethylation of histone H3 at lysine 27 (H3K27) [119]. Extensive studies have demonstrated that EZH2 plays an essential role in orchestrating the functions of T cells, macrophages, and plasma cells [120–123]. EZH2 serves as a negative regulator in NK cells, dampening their effector functions, including cell differentiation, cytotoxic activity, and survival capacity [124].

#### FBP1

FBP1 is a critical rate-limiting enzyme within the gluconeogenesis pathway [125], existing as a homotetramer that transitions between T-state and R-state conformations [126–128]. Within the TME, TGF-β induces the up-regulation of FBP1 expression in tumor-associated NK cells, consequently reducing glycolysis and ultimately driving NK cell functional exhaustion [12].

#### TIPE2

TIPE2 is a critical component of the TIPE protein family [129–131]. The spatial structure of TIPE2 is composed of 6 antiparallel α-helices [132], and its expression is modulated by diverse factors, including TNF-α [105], IL-10 [106], reactive oxygen species (ROS), IL-6, and l-arginine [133]. Studies have demonstrated that TIPE2 acts as a negative modulator of NK cell antitumor immunity; its deletion significantly enhances the cytotoxic activity and cytokine secretion of NK cells, consequently suppressing the progression of solid tumors [106,134].

#### HIF-1α

HIF-1α is a pivotal transcriptional regulator involved in cellular adaptation to hypoxia. Its structure includes an N-terminal basic helix-loop-helix (bHLH) region that dimerizes with the β subunit to execute transcriptional regulation [135–137]. HIF-1α negatively regulates NK cell functionality [138], inhibiting IL-18-mediated nuclear factor κB (NF-κB) signaling pathway activation and attenuating the antitumor activity of tumor-infiltrating NK cells. Studies have shown that HIF-1α inhibitors can robustly enhance the secretion of IFN-γ by human NK cells [12].

## Expression Patterns of NK Cell ICs across Different Tumor Types

Across diverse tumor types, significant heterogeneity exists in the expression profiles of IC molecules on NK cell surfaces, directly influencing their antitumor functionality and immunosurveillance capacity. The dysregulation of the balance between inhibitory and activating IC receptors on NK cells represents a critical mechanism facilitating tumor immune evasion and disease progression. This section systematically summarizes the expression characteristics and regulatory patterns of NK cell IC molecules across various malignancies, with the objective of elucidating the mechanisms through which these molecules influence tumor progression.

### Expression of ICs on NK cells in solid tumors

#### Non-small cell lung cancer

Lung cancer persists as one of the leading causes of cancer-related mortality worldwide, accounting for approximately 25% of all cancer-associated deaths. NSCLC represents the most prevalent histological subtype, constituting approximately 85% of all lung cancer cases and encompasses 3 principal subtypes: adenocarcinoma, squamous cell carcinoma, and large cell carcinoma [97,139–141]. Studies have demonstrated that the frequency of NK cells in the peripheral blood of NSCLC patients is significantly diminished compared to healthy controls [142], potentially compromising antitumor immune responses. Furthermore, the expression levels of ICs on NK cells serve as critical indicators of disease progression. In NSCLC patients, tumor-infiltrating NK cells exhibit significantly elevated PD-1 expression, and these PD-1-positive NK cells concurrently coexpress multiple inhibitory receptors, including TIM-3, TIGIT, KIR2DL3, and KIR3DL1, resulting in compromised NK cell functionality. Studies have demonstrated that blocking the PD-1/PD-L1 axis using specific antibodies effectively inhibits PD-1 aggregation, thereby restoring NK cell functionality and cytolytic activity [143,144]. Additionally, in NSCLC patients, NK cells within the mediastinal lymph nodes exhibit pronounced functional exhaustion, characterized by markedly reduced expression of activating receptors including DNAM-1, NKp46, and NKG2D, as well as diminished activity of the degranulation marker CD107a and decreased production of effector cytokines IFN-γ and TNF-α. Notably, in patients undergoing video-assisted mediastinal lymphadenectomy, PD-1 and CTLA-4 expression on NK cells was significantly diminished, whereas the expression of activating receptors and NKG2A remained predominantly unchanged [97,142].

#### Colorectal cancer

CRC, recognized as the third most prevalent malignancy and the second leading cause of cancer-related mortality worldwide, presents substantial therapeutic challenges predominantly due to delayed diagnosis [145–147]. Studies have demonstrated that TIGIT expression levels on intratumoral NK cells in CRC patients are significantly elevated compared to those on NK cells residing in peritumoral tissues [148]. The TIGIT ligand PVRL2 is highly expressed on tumor cells of patients with colorectal adenocarcinoma, and its interaction with TIGIT strongly correlates with poor clinical outcomes [149]. Elevated levels of granulocyte-monocyte progenitors (GMPs) have been detected in the peripheral blood of CRC patients. These elevated GMP levels contribute to enhanced expression of the granulocyte marker CD15 on monocytes, which subsequently suppresses NK cell activity via the TIGIT and NKp30 signaling pathways. Compared to other IC molecules such as CTLA-4 and PD-1, TIGIT demonstrates a more pronounced association with NK cell functional exhaustion in both murine models and patients with CRC [150].

#### Hepatocellular carcinoma

Chronic hepatitis and cirrhosis resulting from hepatitis C virus (HCV) infection represent major etiological factors for HCC, wherein both innate and adaptive immune responses significantly contribute to HCV-related hepatic injury and disease progression [151]. In HCC patients, both peripheral blood and intrahepatic NK cell populations are markedly depleted, while NK cells within the TME frequently exhibit functional exhaustion, characterized by diminished IFN-γ secretion capacity and impaired cytotoxic activity [78]. Research has demonstrated that TIM-3 and CD38 expression levels on pNKs are substantially elevated in cirrhotic patients, with the expression profiles of these molecules undergoing dynamic modulation during HCC progression [151]. In HCC patients, the homeostatic balance of CD226, TIGIT, and CD96 expression on NK cells is disrupted, characterized by significant up-regulation of CD96, which strongly correlates with elevated TGF-β1 levels in the TME. CD96-positive NK cells exhibit hallmarks of functional exhaustion, including restricted production of pro-inflammatory cytokines (IFN-γ and TNF-α) and compromised cytotoxic function [78,152]. In patients with HBV-associated HCC (HBV-HCC), the NKG2A/NKG2D ratio in NK cells is significantly elevated, both serving as a predictor of tumor progression and correlating positively with NK cell functional suppression. Clinical studies have demonstrated that elevated NKG2A/NKG2D ratios correlate with shortened disease control duration and enhanced immunosuppression in HCC patients [153]. Furthermore, TIGIT and TIM-3 IC molecules are significantly up-regulated on NK cells in HBV-HCC patients, accompanied by substantial impairments in cytotoxic activity and cytokine production capacity [154].

#### Other solid tumors

Numerous studies have documented NK cell IC expression profiles across various solid tumors, including neuroblastoma, gastric cancer, prostate cancer, malignant mesothelioma, head and neck squamous cell carcinoma, and breast cancer [31,87,155–161]. A common characteristic observed in these malignancies is that tumor-infiltrating NK cells exhibit a phenotype characterized by up-regulated inhibitory receptors and down-regulated activating receptors. The interaction between inhibitory receptors and their corresponding ligands leads to functional impairment of tumor-infiltrating NK cells, manifested as diminished ADCC and reduced natural cytotoxicity. These findings provide a compelling rationale for IC blockade (ICB)-targeted therapies, which hold significant promise for reinvigorating NK cell antitumor functionality and improving clinical outcomes.

### Expression of NK cell ICs in hematologic malignancies

#### Acute myeloid leukemia

AML is one of the most common hematologic malignancies in adults, characterized by the excessive growth of immature myeloid progenitor cells within the bone marrow. At present, NK cell-based immunotherapeutic strategies and targeted treatment approaches are undergoing extensive exploration and clinical assessment [76]. Altered NK cell subset distribution implies a potential transition of NK cells from a cytotoxic phenotype to a regulatory phenotype in AML patients [162]. Altered NK cell subset distribution implies a potential transition of NK cells from a cytotoxic phenotype to a regulatory phenotype in AML patients [162]. Compared to healthy donors, AML patients demonstrate elevated CD96 expression levels in both CD3^−^CD56^bright^ and CD3^−^CD56^dim^ NK cell subsets. In newly diagnosed AML patients, CD96^+^ NK cells also demonstrate heightened TIGIT expression, potentially linked to increased IL-10 levels within the TME. Concurrently, these cells exhibit diminished NKp46 and NKG2D expression levels, reflecting an imbalance between activating and inhibitory receptors on NK cells that collectively undermines NK cell functionality, ultimately correlating with adverse clinical outcomes in patients [76,163,164]. AML leukemia cells are characterized by high expression of CD155, which serves as the ligand for TIGIT. Laboratory studies have shown that blockade of the TIGIT signaling pathway can enhance NK cell-mediated anti-leukemia effects [50]. Studies have found that in patients with AML, NK cells expressing TIM-3 exhibit greater responsiveness to stimulation compared to their TIM-3-negative counterparts, and such responses are partially inhibited by TIM-3 blockade. The proportion of TIM-3-positive NK cells in peripheral blood has been shown to enhance prognostic assessments for M1 and M2 (but not M4 or M5) AML patients. This phenomenon may be associated with the absence of a distinct inhibitory signaling motif in the cytoplasmic domain of TIM-3, indicating its potential role as a costimulatory molecule. However, the therapeutic value and prognostic implications of TIM-3 in AML remain underexplored and warrant further investigation [165]. However, the therapeutic value and prognostic implications of TIM-3 in AML remain underexplored and warrant further investigation [165]. Additionally, changes in the levels of other IC receptors have been observed on NK cells in AML patients, notably higher coexpression of TIGIT and PVRIG on CD56^dim^CD16^+^ cells, as well as the up-regulation of NKG2A expression [39,162].

#### Multiple myeloma

MM is a refractory hematological malignancy characterized by abnormal proliferation and accumulation of clonal plasma cells within the bone marrow [166]. Studies have demonstrated that expression of the HAVCR2 gene (encoding Tim-3 protein) is significantly up-regulated in NK cells from newly diagnosed MM patients, while expression levels of other IC molecules remain relatively unaltered. Research has revealed that Tim-3-positive NK cells exhibit significantly reduced expression of functional molecules compared to their Tim-3-negative counterparts, suggesting that Tim-3 negatively regulates NK cell functionality during MM progression. Blocking the Tim-3 pathway in MM models enhances NK cell degranulation and cytotoxic activity against MM cell lines and primary MM cells while simultaneously promoting up-regulation of effector molecules, including perforin and granzyme B. Studies have confirmed that Tim-3 blockade therapy significantly extends survival in MM patients [167].

### Effects of TME factors on the expression of ICs in NK cells

TME represents a highly complex and dynamically evolving ecosystem comprising extracellular matrix, vascular endothelial cells, stromal cells, immune cells, and malignant cells [168,169]. Within the TME, the homeostatic balance between activating and inhibitory molecules on NK cells is profoundly disrupted [170]. Preclinical and clinical studies indicate that NK cell antitumor activity against solid malignancies is governed by multiple factors, with TME-mediated immunosuppression and activation/inhibition imbalances serving as critical determinants of this regulation. Characteristic metabolic perturbations in the TME, including hypoxia, adenosine accumulation, elevated ROS, and increased prostaglandin levels, collectively and profoundly suppress NK cell antitumor functions [171,172].

#### Hypoxic

Hypoxic conditions within the TME of solid tumors are predominantly driven by aberrant vascular architecture and increased oxygen consumption. This hypoxic milieu induces down-regulation of multiple activating receptors (e.g., NKG2D) and effector molecules in NK cells, thereby severely compromising their antitumor activity. Additionally, the hypoxic microenvironment promotes up-regulation of various inhibitory receptors, including co-inhibitory molecules such as KIRs, further attenuating NK cell function [160,161,170,171]. Tumor-infiltrating NK cells typically exhibit profound deficits in cytotoxicity and cytokine secretion capabilities, concomitant with up-regulation of inhibitory receptors such as PD-1 and TIM-3. This functionally exhausted phenotype strongly correlates with elevated levels of immunosuppressive factors within the TME [173]. Furthermore, the TME harbors cell populations that are either recruited or “educated” by malignant cells, including Tregs, stromal cells, M2-polarized macrophages, and myeloid-derived suppressor cells (MDSCs), which collectively compromise NK cell antitumor functions through secretion of immunosuppressive soluble mediators [35,174,175]. Currently, IL-2 preconditioning of NK cells to enhance their adaptability to hypoxic conditions has emerged as a key therapeutic strategy, as studies have demonstrated its efficacy in preventing hypoxia-induced NKG2D down-regulation [171]. ‌Reversing hypoxia represents a pivotal strategy for restoring NK cell functionality within the solid TME. Preclinical evidence indicates that physical exercise may partially alleviate hypoxia [176], while pharmacological inhibition of ERK phosphatase PTPN6 (protein tyrosine phosphatase nonreceptor type 6) effectively prevents hypoxia-driven ERK-STAT3 silencing and consequent NK cell dysfunction. Notably, such interventions may trigger compensatory PD-L1 up-regulation, underscoring the necessity for concurrent ICB strategies [171,177–179]. Future therapeutic developments integrating cytokine-based approaches with IC-targeted modalities hold significant promise for reversing NK cell dysfunction in hypoxic TMEs, potentially transforming the landscape of solid tumor immunotherapy.

#### Metabolic reprogramming in TMEs‌

Tumor cells exhibit markedly enhanced energy metabolism to sustain their proliferative demands. To meet these metabolic requirements, tumor cells up-regulate aerobic glycolysis, a phenomenon characterized as the “Warburg effect”. This metabolic reprogramming enhances nutrient uptake by tumor cells and induces metabolic aberrations of certain substances in the TME (e.g., nucleotides, amino acids, and lipids), simultaneously ensuring efficient energy supply and generation of building blocks necessary for tumor growth while contributing to the suppression of antitumor responses by immune cells [180–184]. Metabolic reprogramming in the TME modulates NK cell effector functions through ICs, whereby tumor-derived lactate or low extracellular pH inhibits NK cell activity by down-regulating the NK activating receptor NKp46 [181,185]. Elevated lactate levels are strongly correlated with enhanced TGF-β activity, which further down-regulates activating receptors (NKG2D, NKp30) and their corresponding ligands [186]. TGF-β antagonizes IL-15-induced mTOR activation, thereby diminishing NK cell function [184]. Tumor cells mediate immunosuppression by metabolizing the essential amino acid tryptophan to kynurenine; furthermore, pro-inflammatory cytokines up-regulate the expression of indoleamine 2,3-dioxygenase [187]. l-Kynurenine has been demonstrated to impair NK cell function by selectively inhibiting the surface expression of NKp46 and NKG2D activating receptors without affecting other surface receptors [188,189]. Although the precise mechanisms linking glycolytic flux and amino acid metabolic dysregulation to NK cell suppression remain incompletely understood, these metabolic imbalances potentially contribute to poor NK cell responses to ICI therapy. Emerging evidence suggests that these metabolic obstacles can be circumvented through combination therapeutic approaches based on ICB [184,190,191].

## Functions and Regulatory Mechanisms of NK Cell ICs

IC molecules expressed on the surface of NK cells serve as critical regulators of their biological functions. Within the TME, malignant cells may compromise the antitumor immune response of NK cells by up-regulating ligands that specifically engage NK cell inhibitory receptors. Simultaneously, interactions between specific IC molecules and ligands on healthy cell surfaces are vital for preserving the functional integrity and homeostasis of NK cells. Therefore, elucidating the regulatory mechanisms and functional attributes of NK cell IC molecules holds great clinical and translational significance for advancing novel NK cell-based immunotherapy strategies [4].

### Molecular mechanisms of ICs in regulating NK cell function

**Fig. 2.**
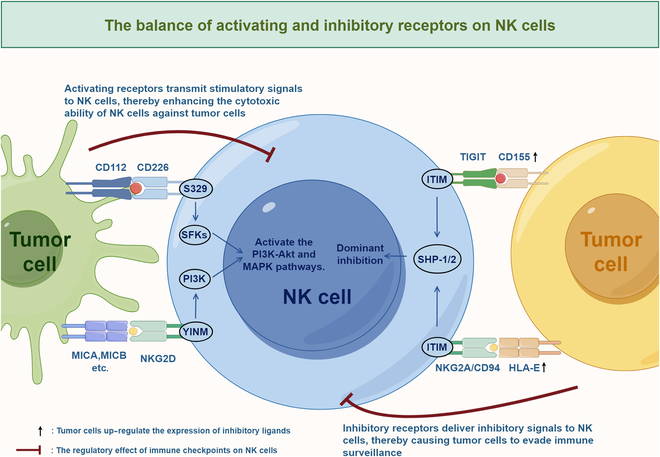
The balance between activating and inhibitory receptors in NK cells. NK cells coexpress both activating and inhibitory receptors, and the balance between these receptors is critical for modulating the antitumor functions of NK cells. These receptors interact with tumor cell surface molecules to either activate or suppress associated signaling pathways, thereby influencing NK cell differentiation, maturation, proliferation, and effector functions. Thus, comprehending the regulatory mechanisms governing these activating and inhibitory receptors, along with their heterogeneity across different tumor types, is pivotal for countering NK cell exhaustion, improving their antitumor potential, and developing innovative cancer therapies. The figure was drawn by Figdraw. ITIM, immunoreceptor tyrosine-based inhibitory motif; SHP, Src homology-2 domain-containing protein tyrosine phosphatase; YINM, tyrosine isoleucine asparagine methionine motif; SFKs, Src family kinases; PI3K, phosphatidylinositol 3-kinase; Akt, protein kinase B; MAPK, mitogen-activated protein kinase; MICA, major histocompatibility complex class I chain-related protein A; MICB, major histocompatibility complex class I chain-related protein B; NKG2D, natural killer group 2 member D.

The functionality of NK cells, pivotal effector components of the innate immune system, is modulated by a complex network of ICs. These checkpoint molecules primarily comprise inhibitory and activating receptors, which precisely regulate the activation state and effector functions of NK cells through distinct signal transduction pathways. Understanding the molecular mechanisms of NK cell ICs is critically important for developing innovative cancer immunotherapy strategies. This section will specifically address the signaling mechanisms of inhibitory receptors in NK cells, the balance between activating and inhibitory receptors, and their functional implications in tumor immunity (Fig. 2).

#### Signal transduction of inhibitory receptors

The functional regulation of NK cells is governed by a series of germline-encoded surface receptors, which transmit either inhibitory or activating signals upon binding to their specific ligands [59]. Inhibitory receptors maintain immune tolerance to self-components by recognizing ligands presented by MHC-I molecules and subsequently suppressing activating receptor-mediated signaling pathways. The cytoplasmic tails of these inhibitory receptors contain ITIMs, which, upon phosphorylation, recruit and activate protein tyrosine phosphatases such as SHP-1 and SHP-2 to mediate inhibitory functions. This inhibitory mechanism is commonly described as “dominant inhibition”. The evasion of NK cell-mediated immune surveillance by tumor cells is predominantly facilitated through the up-regulation of ligands for inhibitory receptors [32]. Most NK cell inhibitory receptors regulate downstream signal transduction through the phosphorylation of ITAMs or ITIMs [142].

#### The balance between activating and inhibitory receptors in NK cells

Accumulating evidence demonstrates that a common phenomenon in various tumors is the down-regulation of NK cell activating receptors and up-regulation of inhibitory receptors, indicating that an imbalance in checkpoint receptor signaling pathways may compromise NK cell functionality [33]. NK cell recognition and response to target cells are contingent upon the fine-tuned regulation of an intricate signaling network involving activating and inhibitory receptors [154]. The balance between activating and inhibitory receptors is crucial for maintaining immune homeostasis, preventing autoimmune diseases, and mitigating immune pathologies associated with infections [192]. In the TME, the down-regulation of NK cell activating receptors and their ligands, coupled with the up-regulation of inhibitory IC molecules, synergistically undermines NK cell antitumor activity and facilitates tumor immune escape [33]. The DNAM-1 and TIGIT/PVRIG/TACTILE signaling axis exemplifies such a mechanism. TIGIT has been shown to demonstrate higher binding affinity for CD155 compared to DNAM-1. Through competitive binding to CD155, TIGIT not only prevents DNAM-1-mediated activating signals but also transmits inhibitory signals to T cells [14,193]. NK cells similarly exhibit such regulatory mechanisms. In HCC, disruption of this signaling balance within and around tumor tissues contributes to the dysfunction of NK cells [37,78]. In ovarian cancer, down-regulation of DNAM-1 and up-regulation of CD96, an inhibitory receptor, lead to the emergence of functionally suppressed and exhausted phenotypes in NK cells. Although recombinant human IL-15 (rhIL-15) can up-regulate the expression of DNAM-1 and TIGIT in a dose-dependent manner, DNAM-1 expression is significantly reduced during coculture of NK cells with ovarian cancer cell line spheroids or patient-derived tumor cells [194]. In individuals with HBV-HCC, research has revealed an imbalance in the expression of NKG2A/NKG2D ICs on NK cell surfaces. This imbalance, characterized by an increased ratio, is strongly correlated with tumor progression and suppresses NK cell function through multiple mechanisms, including reduced cytokine secretion, decreased cytotoxic activity, and increased apoptosis, thereby profoundly influencing patients’ prognosis [153]. Further research has demonstrated the existence of a complex signaling network regulating the NKG2D and DNAM-1/TIGIT receptor systems [96]. Therefore, using specific monoclonal antibodies to block inhibitory signaling pathways could offer a potential strategy for rejuvenating the antitumor capabilities of NK cells [102].

### Aspects of NK cell functions regulated by ICs

**Fig. 3.**
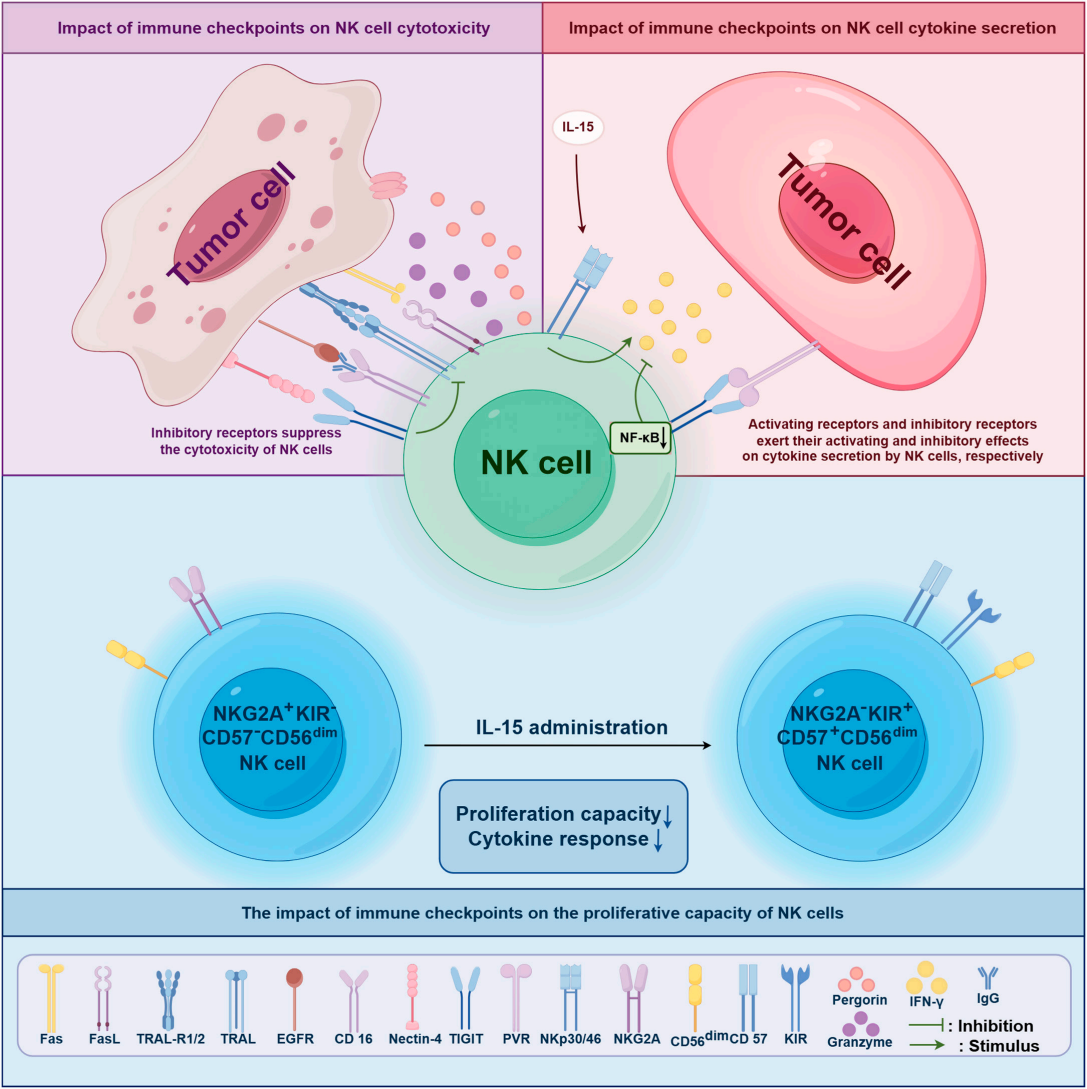
Impact of ICs on the functional activities of NK cells. ICs can modulate NK cell functionality through multiple mechanisms. Inhibitory receptors, such as KIR and NKG2D, down-regulate the NF-κB pathway upon ligand binding, thereby suppressing NK cell-mediated cytotoxicity, cytokine secretion, and proliferation. Consequently, this leads to reduced NK cell activity within the TME, impairing antitumor immune responses and promoting tumor progression. In contrast, activating receptors, such as NKp30, can transmit stimulatory signals to NK cells upon cytokine stimulation, thereby promoting their activation. Tumors evade immune surveillance primarily by up-regulating inhibitory IC molecules. These molecules engage with receptors on the NK cell surface, thereby suppressing their antitumor activity. The figure was drawn by Figdraw. TRAIL, TNF-related apoptosis-inducing ligand; TRAIL-R1/2, TNF-related apoptosis-inducing ligand receptor 1/2; EGFR, epidermal growth factor receptor; Nectin-4, nectin cell adhesion molecule 4; PVR, poliovirus receptor; NKp30/46, natural killer p30/46; KIR, killer immunoglobulin-like receptors; IFN, interferon; IgG, immunoglobulin G.

The functional characteristics of NK cells constitute the cornerstone of their role in mediating antitumor immune responses, which primarily include cytotoxicity, cytokine secretion, and proliferative capacities. These functions are finely regulated by an array of IC molecules, collectively constituting a sophisticated signaling network. Elucidating these regulatory mechanisms and functional characteristics of NK cells is crucial for advancing NK cell-targeted immunotherapeutic strategies. The subsequent section will delve into the functional attributes of NK cells and the regulatory mechanisms governed by IC molecules across these 3 domains (Fig. 3).

#### Cytotoxicity

NK cells possess distinctive immunological properties, demonstrating antitumor cytotoxicity without requiring prior sensitization, while also secreting a range of chemokines and cytokines to modulate immunity [32,195]. Activated NK cells execute their cytotoxic effects through 3 primary mechanisms: (a) degranulation, which involves the release of cytolytic granules containing perforins and granzymes; (b) apoptosis mediated by TNF-related apoptosis-inducing ligand (TRAIL); and (c) apoptosis mediated by Fas ligand (Fas-L) [54]. Among these mechanisms, NK cell-mediated target cell lysis is predominantly accomplished through the secretion of perforins and granzymes from lysosomes, alongside the activation of Fas and TRAIL signaling pathways [196]. NK cell activity is regulated by its surface inhibitory receptors, which modulate NK cell cytotoxicity through interactions with MHC-I molecules displayed on the target cells [97]. Blocking TIGIT molecules on NK cells in primary CRPC cells with the anti-TIGIT monoclonal antibody vibostolimab significantly enhances NK cell in vitro cytotoxicity against primary CRPC cells [54]. TIGIT suppresses NK cell cytotoxicity by specifically recognizing the distal N-terminal IgV domain of nectin-4 via a “lock-and-key” mechanism [197]. Additional studies have confirmed that TIGIT gene knockout enhances NK cell cytotoxicity against diverse cancer cell lines, including spheroids [198]. Additionally, KLRC1 gene deletion has been shown to enhance the cytotoxicity of engineered NKG2AKO NK cells [199]. Regarding activating receptors, the synergistic interaction of DNAM-1 and 2B4 can initiate cytotoxic responses by promoting tyrosine phosphorylation of the adaptor molecule SLP-76 and activating the exchange factor Vav guanine nucleotide exchange factor 1 (Vav-1), thereby counteracting inhibitory receptor signaling [37].

#### Cytokine secretion

NK cells possess the ability to secrete a variety of effector molecules, including cytokines and chemokines. The key cytokines secreted by NK cells include IFN-γ, TNF-α, IL-5, IL-13, and granulocyte-macrophage colony-stimulating factor (GM-CSF). The chemokines secreted by NK cells consist of macrophage inflammatory protein-1 and regulated upon activation normal T cell expressed and secreted [196]. These effector molecules not only enhance the cytotoxic activities of NK cells but also modulate both innate and adaptive immune responses, thus playing a pivotal role in antiviral and antitumor immunity. However, in patients with tumors or chronic infections, the expression and secretion of these molecules are frequently and markedly reduced [97,200]. NK cell activation is finely tuned by the interplay of activating and inhibitory signals, which is key to sustaining their antitumor activity [9]. The inhibitory receptor TIGIT exerts a profound impact on NK cells’ cytokine secretion. Studies have demonstrated that TIGIT expression levels exhibit a significant negative correlation with the ability of NK cells to secrete IFN-γ in patients with cancer and autoimmune disorders [201]. At the molecular level, upon binding to its ligand PVR, TIGIT inhibits IFN-γ production through the NF-κB signaling pathway [200]. In patients with AML, studies have shown that TIGIT-positive NK cells exhibit markedly reduced levels of IFN-γ secretion and cellular degranulation compared with TIGIT-negative NK cells [164]. Beyond TIGIT, the regulatory roles of other IC molecules in modulating NK cell cytokine secretion have also been extensively investigated. Compared with CD96-negative NK cells, CD96-positive NK cells were found to produce markedly lower levels of IFN-γ [76]. PD-1-positive NK cells have been demonstrated to show markedly reduced mean fluorescence intensity of IFN-γ. In patients with active tuberculosis, elevated TIM-3 expression on CD3^−^CD56^+^ NK cells results in suppressed IL-12-induced IFN-γ production [201]. Additionally, in patients with tuberculosis, the CD56^bright^ NK cell subset was found to release substantial levels of cytokines, whereas elevated T cell immunoglobulin and mucin domain protein-1 (TIM-1) expression in this subset impaired NK cell cytokine secretion capacity [202]. Strategies to restore NK cell cytokine secretion functionality are currently under active investigation. Studies have demonstrated that IL-15 activates NK cells through up-regulating the expression of activating receptors NKp30 and NKp46 while down-regulating the inhibitory receptor NKG2A, thereby enhancing NK cell secretion of granzyme B and IFN-γ [90]. Moreover, inhibition of the TIM-3 signaling pathway has been shown to restore NK cell IFN-γ secretion and CD107a expression [201].

#### Proliferative capacity

The biological half-life of NK cells in the human body is approximately 7 days. Under conditions of infection, NK cells enter inflamed tissues from the bloodstream through a cascade process known as “rolling–adhesion–migration” [54]. Once in the tissue, NK cells proliferate in response to activation signals and carry out effector functions, including cytokine secretion and cytotoxicity [104]. The proliferative capacity of NK cells is regulated by various factors, which is significantly influenced by the expression level of the surface marker CD57. Studies have demonstrated that CD57^+^CD56^dim^ NK cells exhibit substantially diminished proliferative capacity compared to their CD57^−^CD56^dim^ counterparts. Furthermore, IC molecules such as NKG2A, KIR, and CD57 have been shown to independently modulate the proliferative potential of CD56^dim^ NK cells [203]. Studies found that NK cell subsets lacking NKG2A expression exhibited variable proliferative capacities depending on KIR expression levels, while NKG2A-positive subsets displayed consistent proliferative potential. These findings underscore the pivotal roles of NKG2A and KIR in maintaining the proliferative potential of NK cells [199]. It has additionally been found that the cytokine IL-21 enhances the expression of activating receptors on NK cells while simultaneously promoting their proliferation [55].

### The relationship between NK cell exhaustion and IC expression

Functional exhaustion of NK cells is vital for the progression of advanced cancer and is primarily characterized by the overexpression of inhibitory receptors on their surface, facilitating tumor cells' evasion of NK cell-initiated immune monitoring [45]. Numerous studies have demonstrated that abnormal levels of inhibitory receptors on NK cells are strongly linked to their functional exhaustion in various pathological conditions, and targeting IC molecules has been shown to effectively reverse this exhaustion. In melanoma patients, NK cell exhaustion is strongly and positively correlated with the overexpression of TIM-3 on their surface, and inhibition of the TIM-3 signaling pathway has been demonstrated to effectively reverse the exhausted phenotype of NK cells, thereby markedly enhancing their antitumor activity [204]. The levels of various IC receptors, including TIGIT and PD-1, is strongly associated with NK cell functional exhaustion in cancer patients [50]. In peritoneal fluid samples from patients with endometriosis, the proportion of PD-1-positive NK cells is markedly elevated, and this immunophenotypic alteration suggests that NK cells may exist in a state of exhaustion. This functional exhaustion may be linked to epigenetic modifications driven by the local microenvironment; however, the precise molecular mechanisms remain to be elucidated and validated [74]. In patients with rheumatoid arthritis, the increased expression of PD-1 on the surface of NK cells is recognized as a key marker of their functional impairment, a phenomenon regulated by microRNAs (miRNAs) [205]. Furthermore, the expression of TIGIT is strongly correlated with NK cell exhaustion in CRC patients and tumor-bearing mice, and targeting the TIGIT signaling pathway in multiple tumor models has been shown to prevent NK cell exhaustion and enhance their antitumor immune responses [148].

### Balance between tumor immune evasion and NK cell functional regulation

Tumor growth and metastasis are regulated by a delicate balance between the host immune system and tumor immune evasion mechanisms. The host immune system recognizes and eliminates malignant cells through its efficient immune surveillance, forming the theoretical foundation of immune-based cancer treatment. Enhancement of the tumor-specific immune response represents a pivotal strategy for inhibiting tumor progression. However, tumor cells gradually acquire immune evasion capabilities through the process of “immune editing”, ultimately escaping clearance by the immune system [57]. Cancer bypass immune monitoring through multiple mechanisms, including the up-regulation of inhibitory receptor ligands [206], the reduction in NK cell recognition ligands, and the secretion of various immunosuppressive factors [2]. Studies have shown that MDSCs, M2 macrophages, and mesenchymal stem cells in the TME facilitate tumor immune escape by secreting immunosuppressive factors, like TGF-β, kynurenine, and prostaglandin E2 (PGE2) [34,207]. The functionality of immune cells is precisely regulated by the activation and inhibitory molecules on their surface. The balance of these signaling pathways is critical for maintaining an effective immune response against pathogens, such as tumors, viruses, and bacteria, while simultaneously preventing autoimmune reactions [22]. Overactivation of inhibitory molecules on NK cells results in functional exhaustion [22], while a reduction in activating receptor expression further facilitates tumor immune evasion [35]. Targeting the interaction between inhibitory receptors, such as TIGIT and NKG2A, and their ligands has the potential to offer novel therapeutic strategies that enhance NK cell antitumor activity and mitigate tumor metastasis [65,208].

## Advances in Preclinical Studies of ICB in NK Cells

### Preclinical models of solid tumors

In recent years, ICB strategies targeting NK cells in solid tumors have shown notable advancements in preclinical research. Research on CRC models has indicated that inhibition of the PVRIG-PVRL2 signaling pathway not only significantly reverses NK cell functional exhaustion but also effectively suppresses tumor progression in the MC38 colon cancer mouse model. Mechanistic studies have elucidated that this therapeutic approach mediates its antitumor effects primarily through the restoration of the effector functions of tumor-infiltrating NK cells and CTLs [149]. Furthermore, studies involving MC38 and other tumor models have revealed that therapeutic strategies targeting TIGIT—including monoclonal antibody blockade, gene knockout, and combination therapies—possess significant therapeutic potential [192,198,209]. For advanced ovarian cancer, researchers have thoroughly investigated the regulatory mechanisms of the DNAM-1/TIGIT/CD96 signaling axis in NK cell-mediated antitumor immune responses. Findings indicate that NK cells derived from patients with advanced ovarian cancer typically exhibit a markedly immunosuppressive phenotype, whereas targeted TIGIT blockade specifically enhances the effector functions of the CD56^dim^ NK cell subset [194]. In lung cancer research, studies have shown that TIM-3 expression is significantly elevated on NK cells in lung adenocarcinoma patients, and anti-TIM-3 blockade effectively restores the cytotoxic functions of these NK cells [210]. Inhibition of the TIGIT pathway sustainably enhances the cytotoxic effects of NK cells against lung cancer cells, thereby offering a crucial theoretical basis for advancing clinical immunotherapeutic strategies [211]. In the PVR-positive A427 lung cancer model, studies have demonstrated that therapeutic strategies targeting KIR2DL5^+^TIGIT^+^ NK cells achieve significantly greater efficacy when employing KIR2DL5 blockade compared to TIGIT blockade [212]. Studies on glioblastoma have indicated that ILT2 blockade restores the tumor-lytic activity of NK cells and augments antitumor immune responses. More specifically, ILT2 blockade, when combined with temozolomide, exhibits significantly enhanced tumor cell clearance effects [87]. Additionally, the monoclonal antibody mAb14-25-9, specifically engineered to target the NKp44-1-PCNA immune complex, demonstrates substantial therapeutic efficacy across various solid tumor models. In recent years, ICB strategies targeting NK cells in solid tumors have shown notable advancements in preclinical research. Research on CRC models has indicated that inhibition of the PVRIG-PVRL2 signaling pathway not only significantly reverses NK cell functional exhaustion but also effectively suppresses tumor progression in the MC38 colon cancer mouse model. Mechanistic studies have elucidated that this therapeutic approach mediates its antitumor effects primarily through the restoration of the effector functions of tumor-infiltrating NK cells and CTLs [149]. Furthermore, studies involving MC38 and other tumor models have revealed that therapeutic strategies targeting TIGIT—including monoclonal antibody blockade, gene knockout, and combination therapies—possess significant therapeutic potential [192,198,209]. For advanced ovarian cancer, researchers have thoroughly investigated the regulatory mechanisms of the DNAM-1/TIGIT/CD96 signaling axis in NK cell-mediated antitumor immune responses. Findings indicate that NK cells derived from patients with advanced ovarian cancer typically exhibit a markedly immunosuppressive phenotype, whereas targeted TIGIT blockade specifically enhances the effector functions of the CD56^dim^ NK cell subset [194]. In lung cancer research, studies have shown that TIM-3 expression is significantly elevated on NK cells in lung adenocarcinoma patients, and anti-TIM-3 blockade effectively restores the cytotoxic functions of these NK cells [210]. Inhibition of the TIGIT pathway sustainably enhances the cytotoxic effects of NK cells against lung cancer cells, thereby offering a crucial theoretical basis for advancing clinical immunotherapeutic strategies [211]. In the PVR-positive A427 lung cancer model, studies have demonstrated that therapeutic strategies targeting KIR2DL5^+^TIGIT^+^ NK cells achieve significantly greater efficacy when employing KIR2DL5 blockade compared to TIGIT blockade [212]. Studies on glioblastoma have indicated that ILT2 blockade restores the tumor-lytic activity of NK cells and augments antitumor immune responses. More specifically, ILT2 blockade, when combined with temozolomide, exhibits significantly enhanced tumor cell clearance effects [87]. Additionally, the monoclonal antibody mAb14-25-9, specifically engineered to target the NKp44-1-PCNA immune complex, demonstrates substantial therapeutic efficacy across various solid tumor models [98].

### Hematological tumor models

In hematological tumor models, NK cell ICB therapies have exhibited significant therapeutic potential and promise for clinical translation. In studies of MM, researchers have engineered a novel PD-1 chimeric switch receptor (PD-1–CSR) specific to NK cells. In-depth investigations have shown that replacing the ITIM and ITSM domains of PD-1 with ITAM or YINM motifs markedly enhances the degranulation and cytokine secretion capacities of NK-92 cell lines and primary NK cells against PD-L1-positive target cells, leading to stronger cytotoxic responses against CD138^+^PD-L1^+^ tumor samples [213]. In AML, TIGIT blockade therapy can effectively reverse TIGIT-mediated suppression of IFN-γ secretion in NK cells while augmenting their cytotoxic activity, thus offering a promising therapeutic target for AML treatment [164]. Specific experimental data demonstrate that single TIGIT receptor blockade significantly improves the 24-hour killing efficiency of NK cells against 3 AML cell lines. Furthermore, blockade of the purinergic signaling pathway through A2AR or CD39 further enhances lysis of AML cells mediated by NK cells. Importantly, dual blockade of A2AR or CD39 in combination with TIGIT exhibits synergistic cytolytic effects in two-thirds of the cell lines, whereas triple blockade markedly enhances NK cell killing efficacy against TF-1 AML cells [162]. In hematological tumor models, NK cell ICB therapies have exhibited significant therapeutic potential and promise for clinical translation. In studies of MM, researchers have engineered a novel PD-1–CSR specific to NK cells. In-depth investigations have shown that replacing the ITIM and ITSM domains of PD-1 with ITAM or YINM motifs markedly enhances the degranulation and cytokine secretion capacities of NK-92 cell lines and primary NK cells against PD-L1-positive target cells, leading to stronger cytotoxic responses against CD138^+^PD-L1^+^ tumor samples [213]. In AML, TIGIT blockade therapy can effectively reverse TIGIT-mediated suppression of IFN-γ secretion in NK cells while augmenting their cytotoxic activity, thus offering a promising therapeutic target for AML treatment [164]. Specific experimental data demonstrate that single TIGIT receptor blockade significantly improves the 24-hour killing efficiency of NK cells against 3 AML cell lines. Furthermore, blockade of the purinergic signaling pathway through A2AR or CD39 further enhances lysis of AML cells mediated by NK cells. Importantly, dual blockade of A2AR or CD39 in combination with TIGIT exhibits synergistic cytolytic effects in two-thirds of the cell lines, whereas triple blockade markedly enhances NK cell killing efficacy against TF-1 AML cells [162].

## CAR-NK Cell Therapy

### Overview of CAR-NK therapy

CAR is an artificially designed fusion protein whose structure is primarily composed of 3 parts: an extracellular antigen-binding domain that recognizes tumor-associated antigens, a transmembrane region, and an intracellular domain that transduces activation signals [214]. To date, researchers have developed multiple generations of CAR structures, significantly enhancing the antitumor activity of T cells and NK cells by optimizing intracellular signaling sequences and integrating various stimulatory and costimulatory domains. Unlike natural TCRs that depend on MHC presentation, CARs can directly recognize antigens on tumor cell surfaces [45,215]. CAR-NK cell therapy employs genetic engineering techniques to express CAR molecules in NK cells, thereby conferring them with the ability to specifically recognize and target antigens on tumor cell surfaces, ultimately resulting in tumor cell elimination through their inherent cytotoxic mechanisms [2]. Based on the unique biological characteristics of NK cells, CAR-NK cell therapy has achieved considerable innovative breakthroughs building upon CAR-T cell therapy, demonstrating not only significant therapeutic advantages but also offering potential alternative treatment options for patients with suboptimal responses to CAR-T cell therapy [7]. Unlike allogeneic T cells that may induce graft-versus-host disease (GVHD), NK cells do not cause GVHD, making CAR-NK cell therapy an ideal therapeutic choice that combines both potent antitumor activity and enhanced safety. The CD3ζ signaling chain is commonly incorporated in CAR-NK cell construction, as it not only enhances the cells’ specific recognition capability for tumor-associated antigens but also improves NK cell expansion efficiency [31]. CAR-NK cells can be derived from allogeneic donors or the NK-92 cell line without requiring strict HLA matching, thereby significantly shortening the interval from patient diagnosis to therapeutic intervention [216]. Compared to CAR-T cells, CAR-NK cells demonstrate significant clinical advantages, particularly their limited in vivo persistence, which effectively reduces the risk of potential damage to normal tissues. Additionally, activated CAR-NK cells primarily secrete IFN-γ and GM-CSF, whereas CAR-T cells secrete predominantly pro-inflammatory cytokines such as TNF-α, IL-1, and IL-6. This significant difference in cytokine secretion profiles further substantiates the safety advantages of CAR-NK cell therapy [45]. Recent research indicates that NK-CAR-induced pluripotent stem cells (iPSCs)-NK cells, developed using induced pluripotent stem cell technology and representing a new generation of engineered immune cells, not only exhibit significant clinical advantages including low toxicity, high response rates, and low recurrence rates but also effectively mitigate the risk of GVHD [7]. Given that NK cells, similar to T cells, express the CD3ζ chain to mediate activation signal transduction, the CD3ζ intracellular domain has become a critical component in NK cell CAR construction. With the advancement of CAR technology, second- and third-generation CAR structures have progressively incorporated costimulatory domains such as CD28 or 4-1BB, further enhancing the functional capacity of these constructs [85].

### Application of CAR-NK cells in cancer treatment

Currently, the limitations of CAR T cells significantly impair their therapeutic efficacy in solid tumors. Therefore, CAR-NK cells represent one of the most promising next-generation CAR immune cells suitable as “off-the-shelf” therapeutic agents [217]. CD19 CAR-NK cells coexpressing IL-15 and CCL-21 [15×21 CAR-NK] exhibit enhanced cytotoxicity and cytokine secretion functions in B cell lymphoma, and have been demonstrated to recruit additional T cells and synergistically eliminate lymphoma cells [218]. Following transduction of the third-generation CAR (CAR-TIM-3) containing TIM-3 single-chain fragment variable (scFv), CD28, 4-1BB, and CD3ζ into human NK-92 cells, these engineered cells effectively recognize and target TIM-3-positive cells, exhibiting significant antitumor activity against various primary AML cell lines, successfully inhibiting in vitro leukemia clone proliferation while exerting minimal impact on hematopoietic stem progenitor cells. Research has demonstrated that engineered NK cells exhibit low TIM-3 expression profiles, which effectively prevents functional exhaustion of NK cells [219]. Researchers have successfully engineered a novel CAR-NK cell subtype—NKG2D-CAR-NK cells. NKG2D-CAR-NK cells, through the incorporation of NKG2D transmembrane domains, specifically mediate NK cell signal transduction, significantly enhancing cytotoxic effects against diverse tumor cell lines, and maintaining robust antitumor activity even within hypoxic TME [56]. Through the development of NKAB-ErbB2 homologous recombinant antibodies, researchers have successfully enabled tumor-specific NKG2D-expressing effector cells to overcome their dependence on membrane-anchored NKG2D ligands, thereby circumventing immune evasion mechanisms [56]. Although CAR-NK cells demonstrate substantial therapeutic potential in tumor treatment, this technology remains in the early developmental stage, confronting multiple technical challenges [45]. These challenges primarily include limited cell expansion capacity, stringent storage condition requirements, susceptibility to Treg-mediated suppression, and attenuated cytotoxic activity due to insufficient cytokine secretion. Additionally, several adverse reactions persist in clinical applications, with the most frequently observed manifestations including fever, fatigue, and anorexia [104].

## Major Challenges and Development Opportunities in NK Cell-Based IC Therapy

**Fig. 4.**
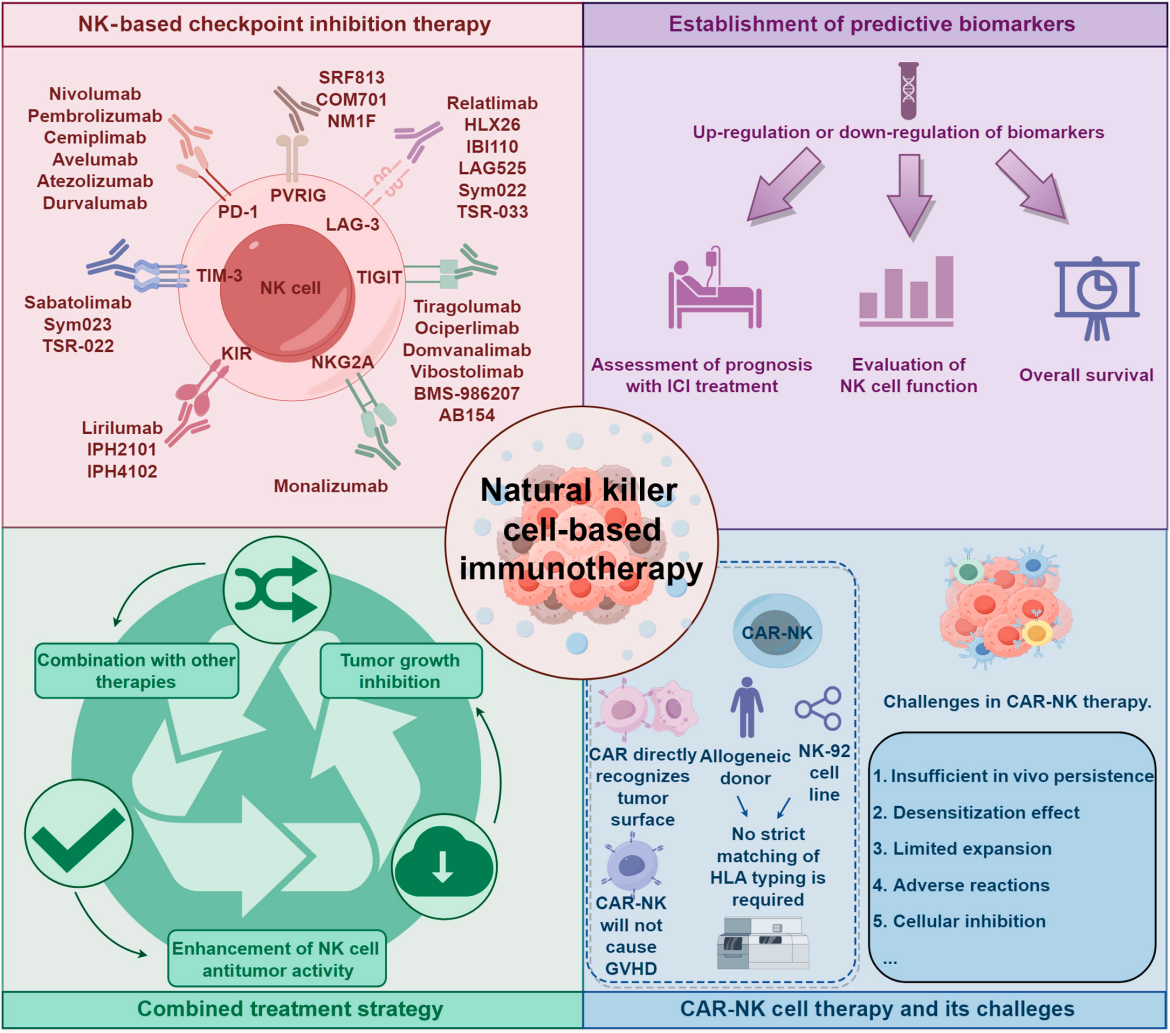
NK cell-based immunotherapies. Currently, numerous immunotherapeutic strategies have been developed with a focus on targeting NK cells. ICIs, whether used as monotherapies or in combination with other immunotherapeutic approaches, have shown promising therapeutic potential in clinical trials. Moreover, the identification and establishment of predictive biomarkers offer critical insights into therapeutic success rates, patient survival outcomes, and the functional evaluation of NK cell-based therapies. CAR-NK cell therapy has also demonstrated significant potential in treating solid tumors. Compared to CAR-T cell therapy, CAR-NK therapy provides distinctive advantages concerning safety and production efficiency. NK cell-based immunotherapies could expand treatment options for tumor-related diseases and are anticipated to play an increasingly pivotal role in the future of cancer therapy. The figure was drawn by Figdraw. PD-1, Programmed cell death protein 1; TIM-3, T cell immunoglobulin domain and mucin domain-3; TIGIT, T cell immunoreceptor with Ig and ITIM domain; LAG-3, Lymphocyte-activation gene 3; PVRIG, Poliovirus receptor-related immunoglobulin domain-containing protein; ICI, IC inhibitors; CAR, Chimeric antigen receptor; GVHD, Graft-versus-host disease.

Despite the substantial advancements in therapeutic strategies targeting NK cell ICs, numerous challenges persist, such as the heterogeneity of NK cell phenotypes and functions, TME-induced NK cell functional exhaustion, and interindividual variations in treatment responses. Nonetheless, these persistent challenges simultaneously unveil new research opportunities within the domain of NK cell immunotherapy. Achieving a comprehensive understanding of the regulatory networks underpinning NK cell IC molecules, optimizing combination therapy regimens, identifying reliable predictive biomarkers, and innovating novel targeted drugs are essential strategies to enhance the clinical efficacy of NK cell immunotherapy while paving the way for personalized precision treatments in the future (Fig. 4).

### Biological challenges

#### Heterogeneity in NK cell phenotypes and functions

Recent research has elucidated that human NK cells exhibit significant heterogeneity in their phenotypes and functions among different individuals, which is associated with specific populations’ susceptibility to various diseases. Evidence has demonstrated that the level of TIGIT on the surface of NK cells in healthy individuals exhibits considerable interindividual variability, and that TIGIT expression is inversely correlated with NK cells’ cytokine secretion capacity and degranulation function [220]. The heterogeneous expression profiles of IC molecules on NK cells across distinct tumor types as well as within individual tumors provide critical guidance for the development of individualized treatment strategies and the assessment of disease prognosis. Using single-cell sequencing technology and flow cytometry, researchers systematically evaluated CD226 (DNAM-1) expression profiles in various tumor tissues and their paired normal tissues. The study demonstrated that CD226 exhibits significant tumor type specificity: Elevated expression is observed in esophageal carcinoma, clear cell renal cell carcinoma, and stomach adenocarcinoma, while lower expression is observed in bladder cancer, chromophobe renal cell carcinoma, thyroid carcinoma, lung squamous cell carcinoma, and HCC [221]. The expression pattern of TIGIT on the surface of NK cells demonstrates considerable variability across diverse disease contexts: In patients with gastrointestinal tumors, NK cells exhibit elevated TIGIT expression alongside diminished IFN-γ production capacity; in contrast, the NK cells of patients with rheumatoid arthritis or systemic lupus erythematosus show decreased TIGIT expression alongside increased capacity for IFN-γ production. The dynamic regulation of NK cell functionality is crucial for maintaining health homeostasis: Impaired NK cell function may increase an individual’s susceptibility to cancer, whereas chronic hyperactivation of NK cell activity may elevate the risk of autoimmune diseases [220].

#### Mechanisms of NK cell dysfunction

NK cells are essential effector cells within the immune surveillance network; however, interactions between their surface inhibitory receptors and tumor cell surface ligands can result in NK cell dysfunction, constituting one of the key mechanisms of tumor immune evasion. Based on this mechanism, restoring the functional activity of NK cells in tumor patients has been identified as a key focus in current tumor immunotherapy research [33]. Studies have demonstrated that TGF-β plays a central regulatory role in inducing NK cell dysfunction. Targeting the TGF-β signaling pathway can effectively restore NK cell metabolic functions and cytotoxic activities, offering a novel therapeutic strategy to optimize NK cell-mediated tumor immunotherapy [170]. Experimental data reveal a significant correlation between elevated TIGIT expression levels and NK cell dysfunction in tumor-bearing mice and CRC patients. In various tumor models, TIGIT blockade not only prevents NK cell dysfunction but also enhances antitumor immune responses through NK cell-mediated mechanisms [148]. Modulating the expression of NK cell activating receptors and their associated factors within the TME represents a promising approach to identify novel therapeutic targets for tumor treatment. Currently, researchers are exploring the use of genetic engineering to transduce cytokine-coding genes into NK cells to potentiate their antitumor immune effects. Although ICIs have shown significant efficacy in certain types of tumors and can partially restore NK cell activity, unlocking the full antitumor potential of NK cells, restructuring the tumor immune microenvironment, and improving treatment outcomes for patients with advanced tumors necessitate the development of more innovative therapeutic strategies [170].

### Future development opportunities

#### Optimization of targeted NK cell therapy combined with other treatments

ICIs have emerged as one of the key strategies in modern cancer immunotherapy approaches. However, large-scale clinical trials have demonstrated significant limitations in the efficacy of monotherapy with ICIs, driving researchers to actively explore innovative therapeutic strategies for nonresponding patients. Immunotherapy targeting NK cells, particularly the application of ICB in combination with other cancer treatment modalities, may represent a promising future direction. Among these efforts, the development of ICI-based multimodal combination therapy strategies has become a major focus in cancer immunotherapy [70]. Previous research has demonstrated that a triple-combination therapy using the anti-NKG2A monoclonal antibody monalizumab, the arginase inhibitor AZD0011, and the anti-PD-1 antibody could significantly improve the complete response rate in tumor-bearing mice, providing critical evidence for clinical translation [222]. In addition, researchers have developed a localized delivery strategy that combines chemotherapy-mediated immunogenic cell death with NK cell-centered ICB therapy. This approach can activate multifaceted immune responses and effectively inhibit tumor growth [223].

#### Establishment of predictive biomarkers

The development of NK cell-related biomarkers has provided precise and reliable molecular diagnostic tools for clinical risk assessment of disease progression and monitoring of therapeutic efficacy [151]. NK cell biomarkers provide insights into cancer prognosis and treatment response by revealing NK cell functional states, thus facilitating tumor identification and elimination through biomarker establishment. Currently established predictive NK cell biomarkers include PD-1/PD-L1, methylated HOXA9, SARIFA, and NKG2A/HLA-E. ICs constitute critical components of predictive biomarkers, which help unravel the intricate interactions and relationships between the immune system and cancer [3]. TIM-3 is constitutively expressed at low levels in resting NK cells, with its expression reflecting IFN-γ-mediated cellular activation states and concurrently acting as a negative regulator of NK cell cytotoxic functions. In patients with HCC, TIM-3 expression on the surface of NK cells is significantly up-regulated. This altered expression pattern suggests that TIM-3 may serve as a specific marker for assessing impaired NK cell functionality [151]. In a bladder cancer study investigating pNKs, researchers explored the expression of KIRs, their HLA class I ligands, and DNAM-1 as potential predictive biomarkers. The findings revealed that specific KIR/HLA–ligand interactions may differentially modulate NK cell-mediated immune surveillance in breast cancer, thereby influencing tumor progression rates and patient survival [224]. In patients with breast cancer, CD56^bright^ NK cells show high expression of TGF-βRII and PD-1, a phenomenon strongly associated with increased plasma levels of sMICA. Studies have shown that tumor-secreted metalloproteinases degrade membrane-bound MICA/B, releasing their soluble forms (sMICA/B) into the bloodstream, where elevated levels are significantly associated with poor patient prognosis [158]. The compositional characteristics of peripheral blood immune cell subsets could serve as potential biomarkers for predicting the prognosis of ICI therapy. Studies have found that the proportion of PD-1^+^CD56^bright^ NK cells in peripheral blood is significantly and negatively correlated with overall survival in patients. This phenomenon may be attributed to the hyperactivation of PD-1-expressing NK cells during ICI therapy, which leads to enhanced secretion of inhibitory cytokines, thereby suppressing the functions of other antitumor immune cells [225]. The research team constructed a predictive model for genes associated with the ratio of NK cells to lymphocytes (NLRs) using the least absolute shrinkage and selection operator–Cox regression (LASSO-Cox) method. The study results demonstrated that the prognostic feature model based on NLRs effectively predicts the clinical responsiveness of melanoma patients to ICB therapy. The NLR feature score was found to be significantly and inversely correlated with the expression levels of inhibitory checkpoint molecules CD274, PDCD1, and CTLA4. Additionally, patients with low NLR scores exhibited longer overall survival and showed better responses to ICB therapy [226]. The establishment of NK cell IC-related predictive biomarkers not only serves as a tool for evaluating tumor progression and therapeutic efficacy but also provides a window into assessing NK cell antitumor functionality and cancer risk stratification. The integration of ICs with other predictive markers, such as ILs and CARs, represents a pivotal direction for elucidating and predicting immunotherapy responses. This approach significantly advances precision medicine by enabling the customization of immunotherapies tailored to personalized biomarker signatures while accounting for the diversity of NK cell responses across cancer types and disease stages. Furthermore, it plays an indispensable role in future tumor immunotherapy development, including the identification of resistance mechanisms and discovery of novel therapeutic targets to enhance treatment efficacy, thereby driving the precision and efficiency of cancer immunotherapies [151,158,224].

## Conclusions

In summary, NK cells represent critical effectors in antitumor immunity, with their function being precisely regulated by IC molecules. This review has comprehensively explored the expression patterns, signaling pathways, and regulatory mechanisms of NK cell ICs involved in tumor immune evasion. The delicate balance between inhibitory and activating checkpoints determines NK cell functional status, with the TME often inducing checkpoint dysregulation that leads to NK cell exhaustion. Therapeutic strategies targeting NK cell ICs, including monoclonal antibodies, small-molecule inhibitors, combination therapies, and advanced approaches such as CAR-NK cells and gene editing, have shown promising results in both preclinical and clinical studies. Despite challenges such as NK cell phenotypic heterogeneity and functional exhaustion, significant opportunities exist for optimizing combination therapies, developing predictive biomarkers, and enhancing personalized treatment approaches to fully harness the antitumor potential of NK cells in cancer immunotherapy.
