# Supplementary material for: Natural Killer Cell Immune Checkpoints and Their Therapeutic Targeting in Cancer Treatment
Source: Research (Wash D C). 2025 Jun 3;8:0723. doi: 10.34133/research.0723 (PMC12131497; doi:10.34133/research.0723)
Supplement: Supplementary 1 — Table S1 [file research.0723.f1.docx]

**Supplementary Table 1. Clinical trials of NK Cell immune checkpoint blockade with pending results.**

|  | National clinical trail no. | Disease | Drugs | Phase | Status |
| --- | --- | --- | --- | --- | --- |
| NKG2A | NCT05221840 | Non-Small Cell Lung Cancer | Durvalumab; Oleclumab; Monalizumab | III | Recruiting |
|  | NCT05061550 | Non-small Cell Lung Cancer | Durvalumab; Oleclumab; Monalizumab | II | Recruiting |
|  | NCT04349267 | Advanced Solid Tumor | BMS-986315; nivolumab; cetuximab | I/II | Terminated |
|  | NCT02643550 | Head and Neck Neoplasms | Monalizumab; Cetuximab; Anti-PD(L)1 | I/II | Completed |
|  | NCT03088059 | Squamous Cell of Head and Neck | Palbociclib; Afatinib | II | Active, not recruiting |
|  | NCT03833440 | Non-small Cell Lung Cancer | Durvalumab; Oleclumab; Monalizumab; | II | Active, not recruiting |
|  | NCT02331875 | Squamous Cell Carcinoma of the Oral Cavity | IPH2201 | I/II | Terminated |
|  | NCT02459301 | Gynecologic Cancer | IPH 2201 | I | Completed |
|  | NCT02921685 | Hematologic Malignancies | Monalizumab | I | Unknown status |
|  | NCT06503614 | Non-muscle Invasive Bladder Cancer | Durvalumab; Monalizumab | II | Not yet recruiting |
| PD-1 | NCT05334329 | Advanced Lung Non-Small Cell Carcinoma | Atezolizumab | I | Active, not recruiting |
|  | NCT05461235 | Gastrointestinal Tumours | Pembrolizumab; Nivolumab; Sintilimab | II | Not yet recruiting |
|  | NCT03383978 | Glioblastoma Multiforme | Ezabenlimab | I | Active, not recruiting |
|  | NCT04847466 | Gastroesophageal Junction Cancers | Pembrolizumab; N-803 | II | Recruiting |
|  | NCT04050709 | Solid Tumor | PD-L1 t-haNK | I | Active, not recruiting |
|  | NCT04390399 | Pancreatic Cancer | PD-L1 t-haNK; N-803; Aldoxorubicin HCl | II | Active, not recruiting |
|  | NCT03228667 | Solid Tumor | N-803; Pembrolizumab; Nivolumab; Atezolizumab | II | Active, not recruiting |
|  | NCT03590054 | Cutaneous Melanoma | Abexinostat; Pembrolizumab | I | Completed |
|  | NCT03815084 | Solid Tumor | pd-1 and DC-NK cells | I | Unknown status |
|  | NCT03841110 | Advanced Solid Tumors | FT500; Nivolumab; Pembrolizumab | I | Completed |
|  | NCT03707808 | Solid Tumor | intratumoral injection of autologous CD1c (BDCA-1)+ myDC | I | Completed |
|  | NCT03958097 | Non-small Cell Lung Cancer | sintiliumab+NK cell | II | Completed |
|  | NCT02913313 | Broad Solid Tumor | BMS-986207; Nivolumab; Ipilimumab | I/II | Completed |
|  | NCT02857166 | Solid Tumors | toripalimab | I | Completed |
|  | NCT03374007 | Advanced Solid Tumor | Geptanolimab | I | Unknown status |
|  | NCT03468751 | Solid Tumor | HLX10 | I | Unknown status |
|  | NCT03474640 | Advanced Malignancies | Toripalimab | I | Unknown status |
|  | NCT02715284 | Neoplasms | Dostarlimab | I | Recruiting |
|  | NCT04414969 | NK/T-cell Lymphoma of Nasal Cavity | Antibody; Peg-Asparaginase; Chidamide | II | Recruiting |
|  | NCT03021057 | T/NK-Cell Lymphoma | pembrolizumab | II | Unknown status |
|  | NCT04602065 | Extranodal NK/T Cell Lymphoma | IBI 318 | I/II | Terminated |
|  | NCT04365036 | NK/T Cell Lymphoma Nos | Toripalimab; Pegaspargase; Gemcitabine | III | Recruiting |
|  | NCT06094296 | Recurrent Non-small Cell Lung Cancer | Nivolumab; BMS-986315; Pemetrexed | II | Completed |
|  | NCT04305054 | Melanoma | Pembrolizumab; Vibostolimab; Quavonlimab | I/II | Recruiting |
|  | NCT05975645 | Advanced Hepatocellular Carcinoma | Penpulimab injection; TQB2618 injection; Anlotinib Hydrochloride Capsules | I | Recruiting |
|  | NCT05034536 | Metastatic Melanoma | Pembrolizumab; Infliximab | II | Recruiting |
| TIGIT | NCT02794571 | Metastatic Tumors | Atezolizumab; Tiragolumab; Carboplatin | I | Active, not recruiting |
|  | NCT04256421 | Small Cell Lung Cance | Tiragolumab; Atezolizumab; Carboplatin | III | Active, not recruiting |
|  | NCT03628677 | Solid Tumor | Domvanalimab; Zimberelimab | I | Active, not recruiting |
|  | NCT05211895 | Non-Small Cell Lung Cancer | Durvalumab; Domvanalimab | III | Recruiting |
|  | NCT02964013 | Neoplasms | Vibostolimab; pembrolizumab; pemetrexed | I | Completed |
|  | NCT03119428 | Locally Advanced Cancer | OMP-313 M32 | I | Terminated |
|  | NCT03260322 | Advanced Solid Tumors | Pembrolizumab; ASP8374 | I | Completed |
|  | NCT01409343 | Solid Tumors | TrasGEX™ | I | Completed |
|  | NCT05394168 | Solid Tumors | HLX 53 | I | Active, not recruiting |
|  | NCT04353830 | Advanced Malignancies | IBI939; Sintillumab | I | Completed |
|  | NCT04354246 | Advanced Cancer | COM 902; COM 701 | I | Recruiting |
|  | NCT04761198 | Advanced Solid Tumor | Etigilimab; Nivolumab | I/II | Completed |
|  | NCT04457778 | Metastatic Solid Tumors | M6223; Bintrafusp alfa | I | Completed |
|  | NCT04543617 | Esophageal Squamous Cell Carcinoma | Tiragolumab; Atezolizumab | III | Active, not recruiting |
|  | NCT06328036 | Recurrent Glioblastoma | Atezolizumab; Tiragolumab | II | Not yet recruiting |
| TIM-3 | NCT03744468 | HNSCC; NSCLC; RCC | Tislelizumab; BGB-A425; LBL-007 | I/II | Recruiting |
|  | NCT03099109 | Solid Tumor | LY3321367; LY3300054 | I | Completed |
|  | NCT02817633 | Neoplasms | TSR-022; Nivolumab; TSR-042 | I | Recruiting |
|  | NCT04139902 | Melanoma Stage | Dostarlimab; TSR-022 | II | Recruiting |
|  | NCT03066648 | Leukemia | Decitabine; PDR001; MBG453; Azacitidine | I | Completed |
|  | NCT05367401 | Acute Myeloid Leukemia | Sabatolimab; Magrolimab; Azacitidine | I/II | Withdrawn |
|  | NCT04266301 | Myelodysplastic Syndromes | Sabatolimab; Azacitidine | III | Completed |
|  | NCT06125652 | Acute Myeloid Leukemia Refractory | anti Tim-3/CD123 CAR-T cell therapy | I/II | Recruiting |
|  | NCT03940352 | Acute Myeloid Leukemia | HDM201; MBG453; Venetoclax | I | Terminated |
|  | NCT04823624 | Myelodysplastic Syndromes | MBG453 | II | Recruiting |
| KIR | NCT01750580 | Solid Tumor | Lirilumab; Ipilimumab | I | Completed |
|  | NCT02252263 | Multiple Myeloma | Elotuzumab; Lirilumab; Urelumab | I | Completed |
|  | NCT01217203 | Multiple Myeloma | IPH 2101; lenalidomide | I | Completed |
|  | NCT02593045 | Cutaneous T-Cell Lymphoma | IPH4102 | I | Completed |
|  | NCT03902184 | Lymphoma | IPH4102 | II | Active, not recruiting |
|  | NCT01256073 | Acute Myeloid Leukemia | IPH2101 | I | Completed |
| LILRB2 | NCT03564691 | Solid Tumor | Pembrolizumab; MK-4830; Carboplatin | I | Active, not recruiting |
|  | NCT06090266 | Solid Tumor | OR502; Cemiplimab | I/II | Recruiting |
|  | NCT06259552 | Solid Tumor | SPX- 303 | I | Recruiting |
| LAG-3 | NCT00349934 | Metastatic Breast Cancer | IMP321 | I | Completed |
|  | NCT03252938 | Solid Tumors | Avelumab; IMP321 | I | Recruiting |
|  | NCT04811027 | Head and Neck Squamous Cell Carcinoma | Pembrolizumab; eftilagimod alpha | II | Active, not recruiting |
|  | NCT02614833 | Adenocarcinoma Breast | IMP321; Paclitaxel | II | Completed |
|  | NCT04095208 | Soft Tissue Sarcoma Adult | Nivolumab; Relatlimab | II | Recruiting |
|  | NCT02676869 | Melanoma | Pembrolizumab; IMP321 | I | Completed |
|  | NCT03642067 | Colorectal Adenocarcinoma | Nivolumab; Relatlimab | II | Active, not recruiting |
|  | NCT03335540 | Advanced Cancer | Nivolumab; Relatlimab; Cabiralizumab | I | Completed |
|  | NCT03704077 | Gastric Cancer | Relatlimab; Nivolumab; Paclitaxel; Ramucirumab | II | Withdrawn |
|  | NCT00351949 | Renal Cell Carcinoma | IMP321 | I | Completed |
|  | NCT05078593 | Solid Tumor | HLX26 | I | Completed |
|  | NCT05101109 | Advanced Solid Tumors | ABL501 | I | Completed |
|  | NCT05400265 | Adult Solid Tumor | HLX26; HLX10 | I | Completed |
|  | NCT06320080 | Advanced Hepatocellular Carcinoma | TQB2223; Penpulimab | I | Not yet recruiting |
|  | NCT03440437 | Advanced Cancer | FS118; Paclitaxel | I/II | Terminated |
|  | NCT06494943 | Head and Neck Squamous Cell Carcinoma | Sintilimab; IBI110; Paclitaxel | I | Active, not recruiting |
|  | NCT05584137 | Colorectal Cancer Metastatic | HLX26; HLX10 | II | Terminated |
| KIR+PD-1+CTLA-4 | NCT03203876 | Advanced Cancer | Lirilumab; Nivolumab; Ipilimumab | I | Completed |
| NKG2A+PD-1 | NCT06116136 | Gastroesophageal-junction Cancer | S095029; pembrolizumab | I/II | Recruiting |
| NKG2A+PD-1 | NCT05162755 | Solid Tumor | S095029; Sym 021; futuximab/modotuximab; anti-HER2 | I | Active, not recruiting |
| PD-1+TIM3+NKG2A | NCT06162572 | Non-small Cell Lung Cancer | S095018; S095024; S095029 | I/II | Recruiting |
| TIGIT+LAG-3 | NCT04150965 | Multiple Myeloma | BMS-986016; BMS-986207; Elotuzumab | I/II | Active, not recruiting |
| PD-1+TIGIT | NCT06250036 | Metastatic Esophageal Cancer,Metastatic Gastric Cancer | Zimberelimab; domvanalimab | II | Not yet recruiting |
| PD-1+TIGIT | NCT04995523 | Non-Small-Cell Lung Carcinoma | AZD2936 | I/II | Active, not recruiting |
| PD-1+TIGIT | NCT05130177 | Melanoma | Zimberelimab; domvanalimab | II | Recruiting |
| PVRIG+TIGIT | NCT05607563 | Advanced Tumor | PM 1009 | I | Unknown status |
| PD-1+TIGIT | NCT05394337 | Metastatic Malignancy | Tiragolumab; Atezolizumab | I/II | Recruiting |
| PD-1+TIGIT | NCT05757492 | Advanced Solid Tumor | CHS-006; toripalimab | I/II | Terminated |
| PD-1+TIGIT | NCT05253105 | Malignancies | TAB006; Toripalimab | I | Withdrawn |
| PD-1+TIGIT | NCT05061628 | Advanced Tumors | JS 006; Toripalimab | I | Unknown status |
| PD-1+TIGIT | NCT05023109 | Biliary Tract Carcinoma | GP; Tislelizumab; Ociperlimab | II | Active, not recruiting |
| PD-1+TIGIT | NCT04656535 | Glioblastoma | AB 154; AB 122 | I | Recruiting |
| PD-1+TIGIT | NCT05019677 | Intrahepatic Cholangiocarcinoma | Tislelizumab; Ociperlimab | II | Withdrawn |
| PD-1+TIGIT | NCT05120375 | Solid Tumor | BAT6021; tislelizumab | I | Terminated |
| PVIRG+TIGIT | NCT04570839 | Solid Tumor | COM701; BMS-986207; Nivolumab | I/II | Completed |
| PD-1+TIGIT | NCT04047862 | Locally Advanced and Metastatic Solid Tumors | Ociperlimab; tislelizumab; Pemetrexed | I | Completed |
| PD-1+TIGIT | NCT05073484 | Advanced Solid Tumor | BAT6021; BAT1308 | I | Terminated |
| PD-1+TIGIT | NCT04693234 | Cervical Cancer | Tislelizumab; Ociperlimab | II | Completed |
| PD-1+TIGIT | NCT05329766 | Gastrointestinal Tract Malignancies | Domvanalimab; Quemliclustat; Zimberelimab | II | Recruiting |
| PD-1+TIGIT | NCT04732494 | Esophageal Squamous Cell Carcinoma | Tislelizumab; Ociperlimab | II | Completed |
| PD-1+TIGIT | NCT05414032 | Locoregionally Advanced Head and Neck Squamous Cell Carcinoma | AZD2936 | II | Recruiting |
| PD-1+TIGIT | NCT04746924 | Non-small Cell Lung Cancer | Tislelizumab; Ociperlimab; Pembrolizumab | III | Active, not recruiting |
| PVRIG+PD-1 | NCT03667716 | Advanced Cancer | COM701; Nivolumab | I | Completed |
| PVRIG+PD-1 | NCT05746897 | Solid Tumor | Pembrolizumab; NM1F | I | Recruiting |
| LAG-3+PD-1 | NCT05645692 | Urothelial Cancer | Atezolizumab; Tobemstomig; Tiragolumab | II | Recruiting |
| CTLA-4+PD-1+TIGIT | NCT05775159 | Hepatocellular Carcinoma,Biliary Tract Cancer | Volrustomig; Bevacizumab; Lenvatinib | II | Recruiting |
| PD-1+TIGIT | NCT04736173 | Non Small Cell Lung Cancer | Zimberelimab; Domvanalimab; Carboplatin | II | Active, not recruiting |
| PD-1+TIGIT | NCT05014815 | Nonsmall Cell Lung Cancer | Ociperlimab; tislelizumab; Pemetrexed | II | Completed |
| PD-1+CTLA-4+TIGIT | NCT04305041 | Melanoma | Pembrolizumab; Quavonlimab; Vibostolimab | I/II | Active, not recruiting |
| PD-1+CTLA-4+TIGIT | NCT05702229 | Gastric Cancer | Rilvegostomig; Volrustomig; FOLFOX | II | Recruiting |
| PD-1+TIGIT | NCT06627647 | Non-squamous Non-small Cell Lung Cancer | Rilvegostomig; Pembrolizumab; Carboplatin | III | Not yet recruiting |
| PD-1+TIM-3 | NCT03680508 | Advanced Adult Primary Liver Cancer | TSR-022; TSR-042 | II | Active, not recruiting |
| PD-1+TIM-3 | NCT04931654 | solid tumors | AZD7789 | I/II | Recruiting |
| PD-1+LAG-3+TIM-3 | NCT03311412 | Solid Tumor | Sym021; Sym022; Sym023 | I | Completed |
| PD-1+TIM-3 | NCT05357651 | Solid Tumor | LB1410 | I | Recruiting |
| PD-1+TIM-3 | NCT03961971 | Glioblastoma Multiforme | MBG453 | I | Active, not recruiting |
| PD-1+TIM-3 | NCT06238635 | Cervical Cancer | Cobolimab; Dostarllimab | II | Recruiting |
| PD-1+TIM-3+LAG-3 | NCT06056895 | Merkel Cell Carcinoma | INCMGA 00012; INCAGN 02385; INCAGN 02390 | II | Recruiting |
| PD-1+CTLA-4 | NCT06608940 | Hepato Cellular Carcinoma (HCC) | BC3402; Durvalumab; Tremelimumab | I/II | Not yet recruiting |
| PD-1+TIM-3 | NCT05216835 | Relapsed or Refractory Classical Hodgkin Lymphoma | Sabestomig (AZD7789) | I/II | Active, not recruiting |
| LAG-3+PD-1 | NCT02658981 | Glioblastoma | BMS986016; nivolumab; urelumab | I | Completed |
| PD-1+LAG-3 | NCT04618393 | Advanced Solid Tumors | EMB-02 | I/II | Terminated |
| PD-1+LAG-3 | NCT03610711 | Gastroesophageal Cancer | Nivolumab; Relatlimab | I/II | Active, not recruiting |
| PD-1+LAG-3 | NCT03005782 | Malignancies | REGN3767; cemiplimab | I | Completed |
| PD-1+LAG-3 | NCT05577182 | Advanced Malignancies | INCA32459-101 | I | Active, not recruiting |
| PD-1+LAG-3 | NCT05645276 | Advanced Malignant Tumors(Stage IA-IB) | AK129 IV | I | Recruiting |
| PD-1+LAG-3+CTLA-4 | NCT04080804 | Head and Neck Squamous Cell Carcinoma | Nivolumab; Relatlimab; Ipilimumab | II | Recruiting |
| PD-1+LAG-3 | NCT03625323 | Head and Neck Squamous Cell Carcinoma,Non-small cell lung cancer | Eftilagimod alpha; Pembrolizumab | II | Active, not recruiting |
| PD-1+LAG-3 | NCT05787613 | Non-small cell lung cancer | Serplulimab; HLX26 | II | Recruiting |
| PD-1+LAG-3 | NCT01968109 | Solid Tumor | Relatlimab; Nivolumab; BMS-986213 | I/II | Active, not recruitinge, not recruiting |
| PD-1+LAG-3 | NCT04140500 | Solid Tumors | RO7247669 | I/II | Active, not recruiting |
| PD-1+LAG-3+TIM-3 | NCT06290622 | Diffuse Large B Cell Lymphoma | Retifanlimab; INCAGN02385; INCAGN02390 | I | Withdrawn |
| PD-1+LAG-3+TIM-3 | NCT04370704 | Melanoma | INCAGN02385; INCAGN02390; INCMGA00012. | I/II | Active, not recruiting |
| PD-1+LAG-3 | NCT05134948 | Advanced Solid Tumors | Relatlimab; Nivolumab | I/II | Active, not recruiting |
| PD-1+LAG-3 | NCT05418972 | Stage II Melanoma | Relatlimab; nivolumab | II | Recruiting |
| PD-1+LAG-3 | NCT05498480 | Advanced Solid Tumors | Relatlimab; nivolumab | I | Completed |
| PD-1+LAG-3 | NCT03219268 | Advanced Solid Tumors,Hematologic Neoplasms | tebotelimab | I | Completed |
| PD-1+LAG-3+CTLA-4 | NCT05704933 | Metastatic Melanoma | Nivolumab; Ipilimumab; Relatlimab | I | Active, not recruiting |
| PD-1+LAG-3 | NCT03607890 | MSI-H Tumors | Relatlimab; Nivolumab | II | Recruiting |
| PD-1+LAG-3 | NCT05352672 | Melanoma | Fianlimab; Cemiplimab; Pembrolizumab | III | Recruiting |
| PD-1+LAG-3 | NCT06288191 | Cutaneous Squamous Cell Carcinoma | Nivolumab; Relatlimab | II | Not yet recruiting |
| PD-1+LAG-3 | NCT04658147 | Hepatocellular Carcinoma | Nivolumab; Relatlimab | I | Recruiting |
| PD-1+LAG-3 | NCT02966548 | Solid Tumor | Relatlimab; Nivolumab | I | Active, not recruiting |
| PD-1+LAG-3 | NCT03743766 | Melanoma | Relatlimab; Nivolumab | II | Completed |
| PD-1+LAG-3+CTLA-4 | NCT03459222 | Advanced Cancer | Relatlimab; Nivolumab; Ipilimumab; BMS-986205 | I/II | Active, not recruiting |
| PD-1+LAG-3+TIM-3 | NCT04785820 | Advanced or Metastatic Esophageal Squamous Cell Carcinoma | Lomvastomig; Tobemstomig; Nivolumab | II | Active, not recruiting |
| PD-1+LAG-3 | NCT05608291 | Melanoma | Fianlimab; Cemiplimab; Pembrolizumab | III | Recruiting |
| PD-1+LAG-3+CTLA-4 | NCT06295159 | Melanoma | Nivolumab; Relatlimab; Ipilimumab | I | Recruiting |
| PD-1+LAG-3 | NCT06151236 | Merkel Cell Carcinoma | Nivolumab; Relatlimab | II | Recruiting |
| PD-1+LAG-3 | NCT06036836 | Solid Tumor | Favezelimab; pembrolizumab; lenvatinib | II | Recruiting |
| PD-1+CTLA-4 | NCT06264180 | Advanced Melanoma | Nivolumab; Relatlimab | III | Recruiting |
| PD-1+LAG-3 | NCT06246916 | Melanoma | Relatlimab; nivolumab; fianlimab; cemiplimab | III | Recruiting |
| PD-1+LAG-3 | NCT05800015 | Non-small Cell Lung Cancer | Fianlimab; Cemiplimab | II/III | Recruiting |
| PD-1+LAG-3 | NCT03044613 | GastroEsophageal Cancer | Nivolumab; Relatlimab; Carboplatin | I | Active, not recruiting |
| PD-1+LAG-3 | NCT06571708 | Bladder Cancer | Gemcitabine; Cisplatin; Cemiplimab; Fianlimab | II | Not yet recruiting |
| PD-1+LAG-3 | NCT06586294 | Gastroesophageal Junction Adenocarcinoma | AK129; oxaliplatin; capecitabine | I/II | Not yet recruiting |
| PD-1+LAG-3 | NCT03623854 | Chordoma | Nivolumab; Relatlimab | II | Completed |
| PD-1+LAG-3 | NCT05785767 | Advanced Non-Small Cell Lung Cancer | Fianlimab; cemiplimab | II/III | Recruiting |

**Abbreviations:** NKG2A: Natural Killer Group 2 Member A; PD-1: Programmed Death-1; TIGIT: T-cell immunoreceptor with immunoglobulin and immunoreceptor tyrosine-based inhibitory motif domains; TIM-3: T cell immunoglobulin and mucin-domain containing-3; KIR: Killer Cell Immunoglobulin-Like Receptor; LAG-3: Lymphocyte Activation Gene-3; CTLA-4: Cytotoxic T Lymphocyte Antigen-4
